# Hydrogenated silicene nanosheet functionalized scaffold enables immuno‐bone remodeling

**DOI:** 10.1002/EXP.20220149

**Published:** 2023-05-28

**Authors:** Zixuan Lin, Zhixin Chen, Yiwei Chen, Nan Yang, Jianlin Shi, Zhongmin Tang, Changqing Zhang, Han Lin, Junhui Yin

**Affiliations:** ^1^ Institute of Microsurgery on Extremities Department of Orthopaedic Surgery Shanghai Sixth People's Hospital Affiliated to Shanghai Jiao Tong University School of Medicine Shanghai P. R. China; ^2^ State Key Laboratory of High Performance Ceramics and Superfine Microstructure Shanghai Institute of Ceramics Chinese Academy of Sciences Shanghai P. R. China; ^3^ Shanghai Tenth People's Hospital Shanghai Frontiers Science Center of Nanocatalytic Medicine School of Medicine Tongji University Shanghai P. R. China; ^4^ Departments of Radiology and Medical Physics University of Wisconsin‐Madison Madison Wisconsin USA

**Keywords:** biodegradation, bone remodeling, osteoimmunomodulation, silicene nanosheet

## Abstract

An ideal implant needs to have the ability to coordinate the foreign body response and tissue regeneration. Here, Hydrogenated‐silicon nanosheets (H‐Si NSs) with favorable biodegradability are integrated and functionalized into a β‐tricalcium phosphate scaffold (H‐Si TCP) for bone defect healing. H‐Si TCP can greatly improve bone regeneration through osteoimmunomodulation‐guided biodegradation in vivo. The spatiotemporal regulation of degradation products replenishes sufficient nutrients step by step for the entire process of bone repair. Extracellular and intracellular reactive oxygen species (ROS) are first downregulated by reaction with H‐Si NSs, followed by marked M2 polarization, remodeling the micro‐environment timely for immune‐bone regeneration. The release of primary reaction products awakened bone marrow mesenchymal stem cells (BMSCs), which are converted into osteoblasts anchored on scaffolds. Subsequently, biomineralization is promoted by the final degradation products. The intrinsic ROS‐responsive, immunoregulatory, and osteo‐promotive capability of 2D H‐Si NSs makes such composite H‐Si TCP scaffold a highly potential alternative for the treatment of critical bone defect.

## Graphical abstract

A novel 2D hydrogenated‐silicon nanosheet (H‐Si NSs) is integrated and functionalized into a β‐tricalcium phosphate scaffold (H‐Si TCP) for immuno‐bone remodeling in our study. H‐Si NSs has great ROS‐responsive, immunoregulatory, and osteo‐promotive property and H‐Si TCP can provide sufficient ingredients and greatly improve bone regeneration step by step through osteoimmunomodulation‐guided biodegradation in vivo.

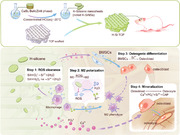

## 1 INTRODUCTION

Although bone displays strong self‐healing property upon injury, repairing bone defects of critical size remains a great clinical challenge. Generally, traditional bone defect management includes bone transplants, either through autografts or allografts. However, the concerns of limited supply, donor site morbidity, as well as risks of infection and disease transmission have greatly restricted the application of these natural grafts in bone healing.^[^ ^1^ ^]^ To address these limitations, various synthetic bone substitutes have emerged as alternatives to restore the normal function of bone injury. In fact, bone grafting procedures have gradually shifted from natural grafts to synthetic substitutes in the last few years and become a routine regenerative procedure in clinical practice.^[^ ^2^ ^]^ However, due to the complex interplays between skeletal and immune system, the implanted materials in favor of osteogenesis in vitro may actually cause a series of undesired host responses in vivo such as foreign body response and massive oxidant formation through the constant oxidative attack by immune cells. The excessive or prolonged oxidative exposure could result in chronic and dysregulated inflammation, extensive fibrosis as well as the loss of biocompatibility and function of materials, and profoundly undermine the functional bone regeneration.^[^ ^3^ ^]^ As a result, the research and development of biomaterials with effective oxidant‐regulatory property could be a promising strategy to improve the bone healing outcomes.^[^ ^4^ ^]^

The development of nanomedicine has made great progress in terms of orthopedic therapies.^[^ ^5^ ^]^ Compared with bulk materials, nanomaterials enjoy the advantages of good biocompatibility, easy metabolism, minor side effects, good targeting, and improved therapeutic efficacy.^[^ ^6^ ^]^ Silicon is indispensable in bone biology and extensively used in bone biomaterials. However, the conventional silicon‐based nanoparticles were limited in clinic applications due to their poor biodegradability induced by implantation.^[^ ^7^ ^]^ By introducing the surface covalent bonding of hydrogen atoms on metastable two‐dimensional (2D) silicene, we constructed brand new two‐dimensional hydrogen‐bonded silicene nanosheets (H‐Si NSs) in our previous research,^[^ ^8^ ^]^ with feature of physiochemical nature for photo‐triggered therapeutics as other 2D nanomaterials.^[^ ^9^ ^]^ Especially, the H‐Si NSs displayed greatly favorable effects of biocompatibility and biodegradation. Recently, we found that the 2D H‐Si NSs developed in our lab could timely respond and smartly modulate the concentrations of reactive oxygen species (ROS) especially in the context of significant oxidative stress. Among all the synthetic bone substitutes available, calcium phosphate‐based biomaterials are most widely used in orthopedic practice.^[^ ^10^ ^]^ As the major component in bone tissue, calcium phosphate is of great biocompatibility and able to provide a rich source of calcium and phosphorus for bone biomineralization.^[^ ^11^ ^]^

**SCHEME 1:**
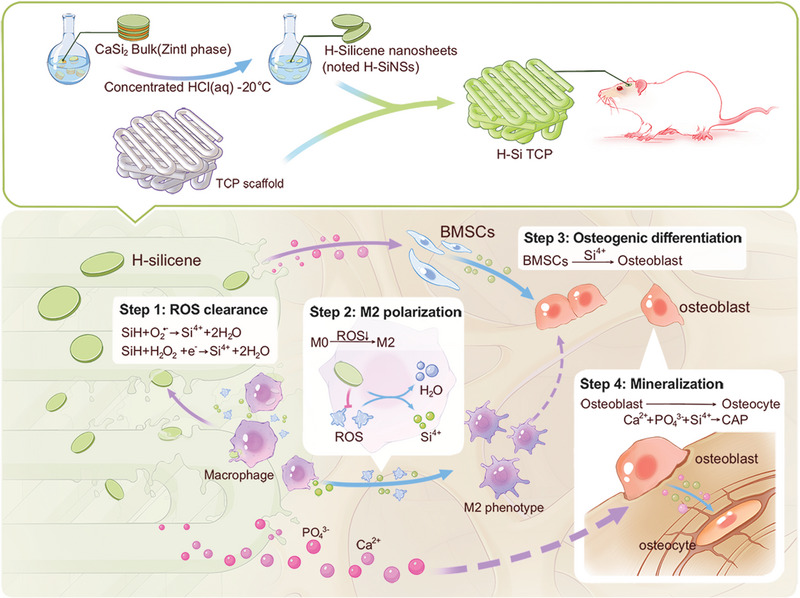
The fabrication of H‐Si TCP scaffolds and mechanism of H‐Si NSs integrated β‐TCP in bone defect remodeling. H‐Si TCP drives bone remodeling step by step through osteoimmunomodulation coordinated with biodegradation. TCP, tricalcium phosphate.

Herein, the 2D H‐Si NSs were integrated into a 3D‐printed bone‐mimetic β‐tricalcium phosphate (TCP) as a versatile H‐Si TCP scaffold. In this composite, β‐TCP is highly biocompatible and osteoconductive, and provides a resorbable interlocking network and support. H‐Si TCP has advanced the innovation of implants for the orchestration of scaffold degradation and bone regeneration through osteoimmunomodulation. In other words, H‐Si TCP could remodel the local immune microenvironment and supplement sufficient mineral products following the spatiotemporal regulations of scaffold degradation step by step for the whole process of bone repair (Scheme 1). To be more specific, H‐Si NSs were first released from the composite scaffold and scavenged the overproduction of local environmental ROS (Step 1). In the meantime, H‐Si NSs were endocytosed into macrophages and downregulated their intracellular ROS levels, facilitating M2 polarization (Step 2). Consequently, the pro‐inflammatory immune microenvironment was converted into anti‐inflammatory type, orchestrating the scaffold degradation and bone regeneration. On one hand, the ionic silicon derived from silicon nanosheets was released and promoted osteogenic differentiation (Step 3). On the other hand, the primary degradation products of H‐Si TCP scaffold facilitated osseous tissue mineralization to realize functional bone repair (Step 4). What's more, mineralization could trigger further dissociation of H‐Si TCP scaffold, and the entire degradation process in turn would provide more space available for new bone tissue formation. To sum up, H‐Si TCP composites developed in our study enables immuno‐bone remodeling to facilitate bone regeneration, and thus could be a promising substitute to fix large bone defects.

## 2 RESULTS AND DISCUSSION

### 2.1 Synthesis and characterization of H‐Si nanosheets

**FIGURE 1:**
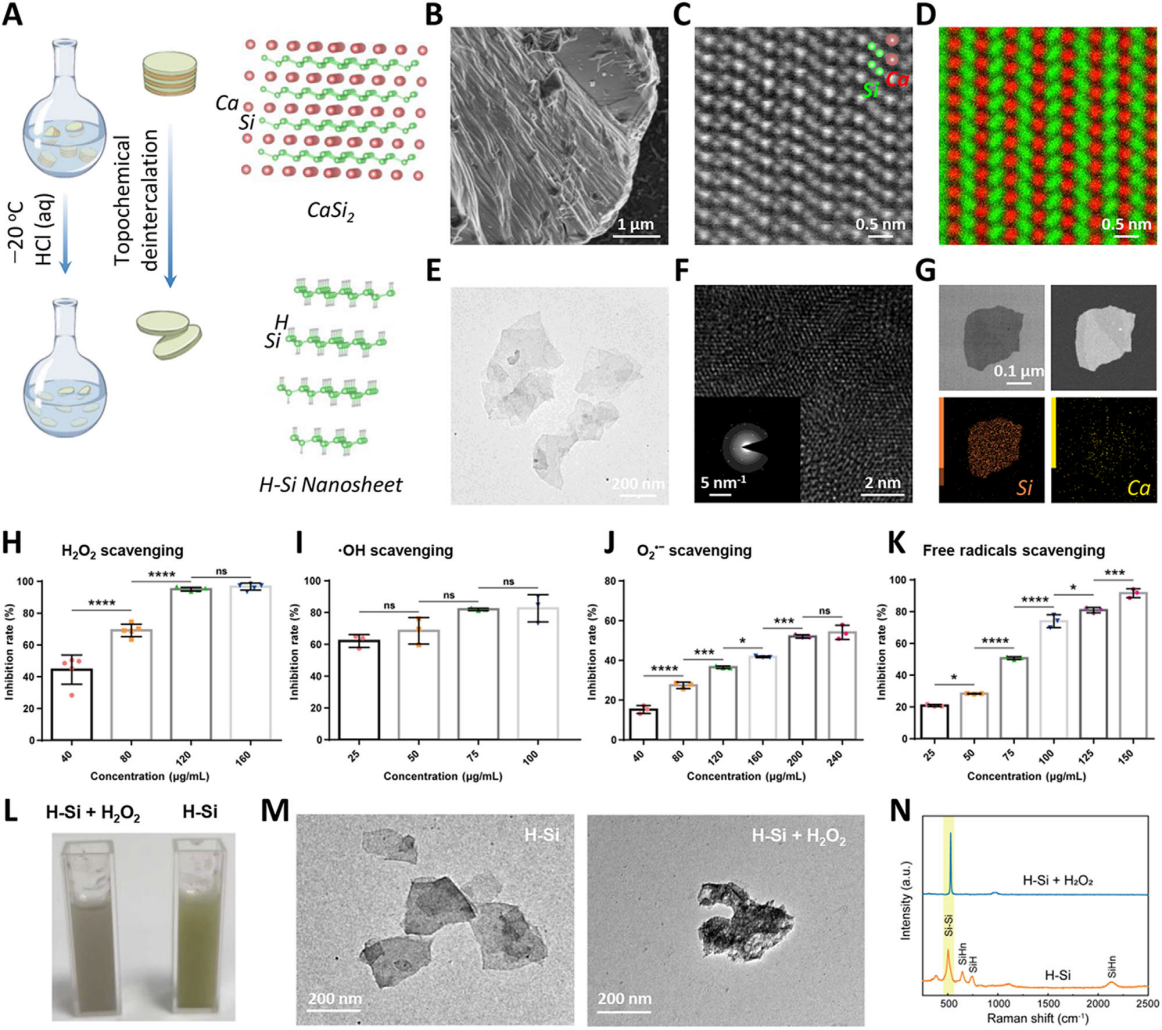
Synthesis and characterization of H‐Si NSs. A) Scheme for the topochemical deintercalation process of 2D H‐Si NSs and the corresponding ball‐and‐stick models. B) Scanning electron microscopy (SEM) image of CaSi_2_ bulk. C) Aberration‐corrected high‐angle annular dark‐field scanning transmission electron microscopy (HAADF‐STEM) images of H‐Si NSs exhibiting the bilayer sandwich calcium and silicon atoms as bright dots. D) Elemental mapping of pristine CaSi_2_. E) Transmission electron microscopy (TEM) image, F) high resolution transmission electron microscopy (HR‐TEM) image, and selected area electron diffraction (SAED) pattern of the H‐Si NSs. G) Bright‐field and dark‐field TEM images and the corresponding elemental mapping of the certain H‐Si NSs. The evaluations of H‐Si NSs in scavenging H) H_2_O_2_, I) •OH, J) O_2_ •−, and K) ABTS^+•^. L) Photographs, M) TEM images, and N) Raman images of H‐Si NSs before and after receiving the treatments of H2O2. ^****^ *p* < 0.0001; ^***^ *p* < 0.01; ^*^ *P* < 0.05; ns, no significance, one‐way ANOVA. ROS, reactive oxygen species; BMSCs, bone marrow mesenchymal stem cells; TCP, tricalcium phosphate.

The 2D H‐Si NSs were synthesized by an oxidative etching of Zintl phase CaSi_2_ using hydrochloric acid (HCl) via topochemical deintercalation mechanism, which is a modified method based on our previous reports.^[^ ^8^ ^]^ Briefly, the CaSi_2_ compound was subject to an oxidation process in pre‐cooled aqueous hydrochloric acid medium via selective oxidation and etching (Figure 1A). Scanning electron microscopy (SEM) image demonstrated that the CaSi_2_ bulk presented a typical microstructure of the layered binary silicide, promising the selective Si nanolaminate extraction from the parent CaSi_2_ (Figure 1B). The aberration‐corrected high‐angle annular dark‐field scanning transmission electron microscopy (AC HAADF‐STEM) image manifested that the buckled honeycomb [Si]_n_^−^ atom layers were separated by the [Ca]_n_^+^ atom layers in atomic resolution (Figure 1C). The corresponding elemental mapping further indicated that these alternating 2D H‐Si buckled sheets were isolated by planar sheets of Ca (Figure 1D). Based on the layered intermetallic “Zintl” phase precursor of CaSi_2_ consisting of periodic metallic Ca layers and corrugated Si layers (Figure 1A), the Ca atom layers were etched by HCl by an outer‐inner diffusion‐controlled process, and the final precipitates were H‐Si NSs. Transmission electron microscopy (TEM) image showed that the synthesized H‐Si NSs were ultrathin and nanoscale‐lateral size, which indicated the typical 2D free‐standing morphology (Figure 1E). The high resolution transmission electron microscopy (HR‐TEM) image exhibited the relative complete crystallization of H‐Si NSs with hexagonal structure, and the corresponding selected area electron diffraction (SAED) pattern also featured the preserved hexagonal phase (Figure 1F). The elemental mapping of H‐Si NSs clearly demonstrated the effective Ca‐removal from calcium silicide precursor (Figure 1G).

The representative ROS as H_2_O_2_, ·OH and O_2_^•−^ were chosen to investigate the ROS‐scavenging antioxidative capacity of 2D H‐Si NSs. The dose‐dependent ROS scavenging activities of H‐Si NSs were qualitatively and quantitatively examined. We first assessed the consumption rates of H‐Si NSs against H_2_O_2_ (Figure 1H). It could be found that a 90% H_2_O_2_‐inhibition rate of H‐Si NSs at the concentration of 120 µg mL^−1^ had been collected. In similar, ·OH could be depleted exceeding 80% by H‐Si NSs at a relatively low concentration of 75 µg mL^−1^ (Figure 1I). Additionally, an effective O_2_^•−^‐inhibition rate near 60% by H‐Si NSs had been obtained at the concentration of 200 µg mL^−1^ (Figure 1J). It was noted that a significant positive correlation was found between the clearance rate of O_2_^•−^ the concentration of H‐Si NSs. To further determine the total antioxidant capacity of H‐Si NSs, the total free radicals scavenging assay was measured by the standard ABTS (2, 2′‐azinobis (3‐ethylbenzothiazoline 6‐sulfonate)) radical cation decolorization assay. As depicted in Figure 1K, the H‐Si NSs induced reduction of ABTS^+•^ radicals contributed to a marked elimination (∼ 80%) of free radicals at an H‐Si NSs concentration of 100 µg mL^−1^. Therefore, it is envisaged that H‐Si NSs possess the strong capability of detrimental ROS‐scavenging in vitro. In addition to a robust ROS‐scavenging ability, H‐Si NSs exhibited ROS‐responsive degradation behavior that once encountering free radicals, typically like H_2_O_2_, discoloration occurred (Figure 1L). The TEM images showed that the lamellar structure of H‐Si NSs was obviously damaged after the function as the scavenger of H_2_O_2_ (Figure 1M). Furthermore, the disappearance of Raman characteristic peaked before and after the scavenging reaction was also a good indication of the destruction of the H‐Si NSs structure and the occurrence of degradation behavior (Figure 1N). The infrared spectrum also confirmed the disappearance of the characteristic peak belonging to Si─H bond after the scavenging reaction (Figure S1).

### 2.2 H‐Si TCP showed great biocompatibility in vitro

**FIGURE 2:**
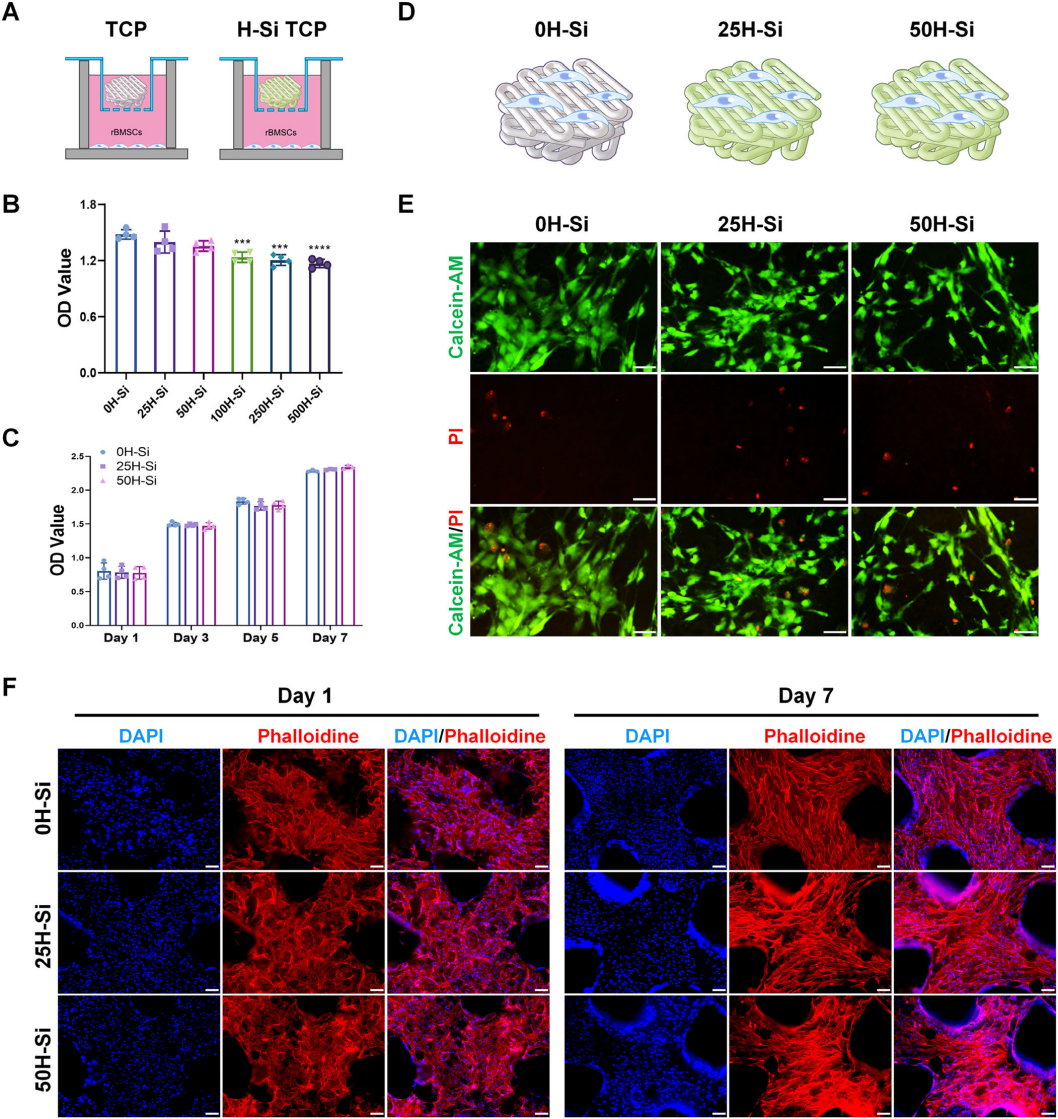
Biocompatibility of tricalcium phosphate (TCP) and H‐Si TCP scaffold in vitro. A) Schematic diagram of bone marrow mesenchymal stem cells (BMSCs) incubated with TCP scaffold integrated with different concentrations of H‐Si NSs. B) The CCK‐8 assay of BMSCs incubated with TCP scaffold integrated with different concentrations of H‐Si NSs for 24 h. *N* = 4. ^***^ *p* < 0.001, ^****^ *p* < 0.0001 (Significant difference in comparison with 0H‐Si). C) The CCK‐8 assay of BMSCs incubated with TCP scaffold integrated with 0, 25, and 50 µg mL^‐1^ H‐Si NSs at days 1, 3, 5, and 7. No significant difference was observed between different groups at the same timepoint. D) Schematic diagram of BMSCs seeded onto TCP, 25H‐Si TCP, and 50H‐Si TCP scaffold. E) Calcein‐AM/PI co‐staining of BMSCs grown on TCP, 25H‐Si TCP, and 50H‐Si TCP scaffold was performed at day 1. The green fluorescence labeled live cells and the red fluorescence labeled dead cells. Bar = 50 µm. F) The 4′,6‐diamidino‐2‐phenylindole (DAPI)/Phalloidin staining of BMSCs cultured on TCP, 25H‐Si TCP, and 50H‐Si TCP scaffold at days 1 and 7. The nuclei were stained with DAPI in blue fluorescence, and the cytoskeleton was stained with rhodamine phalloidin in red fluorescence. Bar = 100 µm.

The TCP scaffolds were fabricated via three‐dimensional (3D) printing technique and the H‐Si TCP scaffolds were constructed by integrating H‐Si NSs into the TCP scaffolds via a simple surface modification method in our experiments.^[^ ^12^ ^]^ To assess the biocompatibility of scaffolds in vitro, bone marrow mesenchymal stem cells (BMSCs) were first incubated with H‐Si TCP scaffolds loaded with different concentrations of H‐Si NSs for 24 h to assess the cytotoxicity. TCP scaffold was set as control (Figure 2A). As the result shows, 25H‐Si TCP and 50H‐Si TCP did not exhibit significant cytotoxicity to BMSCs after 24 h of co‐culture. When the concentration of H‐silicene reached or exceeded 100 µg mL^−1^, the growth of BMSCs was significantly inhibited (Figure 2B). BMSCs were then incubated with 25H‐Si TCP and 50H‐Si TCP for 7 days to assess their cytotoxicity at longer exposure. Cell Counting Kit‐8 (CCK‐8) assay revealed that 25H‐Si TCP and 50H‐Si TCP did not exhibit significant adverse effects on BMSCs growth and proliferation at days 1, 3, 5, and 7 (Figure 2C). Thus, 25H‐Si TCP and 50H‐Si TCP scaffold were used in the following in vitro studies.

Then, BMSCs were seeded onto the TCP, 25H‐Si TCP, and 50H‐Si TCP to further assess the cytocompatibility of these scaffolds (Figure 2D). Cell viability of BMSCs on scaffolds was co‐stained by calcein acetoxymethyl ester (calcein‐AM) and propidium iodide (PI) on day 1. To be specific, live cells were stained in green fluorescence and dead cells were stained in red fluorescence. As shown in Figure 2E, the great majority of BMSCs were stained in green, indicating most of the cells were alive on both composite scaffolds. No significant difference was seen between different groups in live/dead cells ratio (Figure S2). Meanwhile, the cell adhesion and proliferation of BMSCs on these scaffolds were examined on days 1 and 7. 4′,6‐diamidino‐2‐phenylindole (DAPI) and rhodamine phalloidin staining were performed to visualize the nuclei and cytoskeletons of BMSCs. On days 1 and 7, BMSCs were attached to the surface of scaffolds with well‐spread morphology and abundantly extended pseudopods, indicating great cell adhesion and vitality on all these scaffolds. No significant difference in cell count was seen between different groups at days 1 and 7 respectively (Figure S3). Besides, the cell number of BMSCs cultured on TCP, 25H‐Si TCP, and 50H‐Si TCP scaffolds multiplied at day 7, suggesting their great capacity for cell proliferation (Figure 2F). To summarize, the results above demonstrated the high biocompatibility of TCP, 25H‐Si TCP, and 50H‐Si TCP scaffolds, which offered desirable supports for BMSCs attachment, growth, and proliferation.

### 2.3 H‐Si TCP upregulated M2 macrophage polarization

Due to the high plasticity and heterogeneity, macrophages were deemed as the model cell type and prime target in the modulation of immune response including osteoimmunomodulation.^[^ ^3b^ ^]^ M2 type macrophage was considered to play critical role in the resolution of inflammation and progression of tissue repair,^[^ ^13^ ^]^ and the downregulation of intracellular ROS was potentially implicated in M2 macrophage polarization.^[^ ^14^ ^]^ Since H‐Si NSs could scavenge excessive extracellular ROS under inflammation (Figure 1H‐K), the chances are great that the H‐Si NSs released from composite scaffolds would downregulate intracellular ROS level as well when endocytosed by surrounding macrophages and regulate their polarization. As the result, we assumed that H‐Si TCP scaffolds were able to facilitate M2 macrophage polarization. The combination of lipopolysaccharide (LPS) plus Interferon‐γ (IFN‐γ) was utilized to simulate inflammatory status in bone defect upon injury.

**FIGURE 3:**
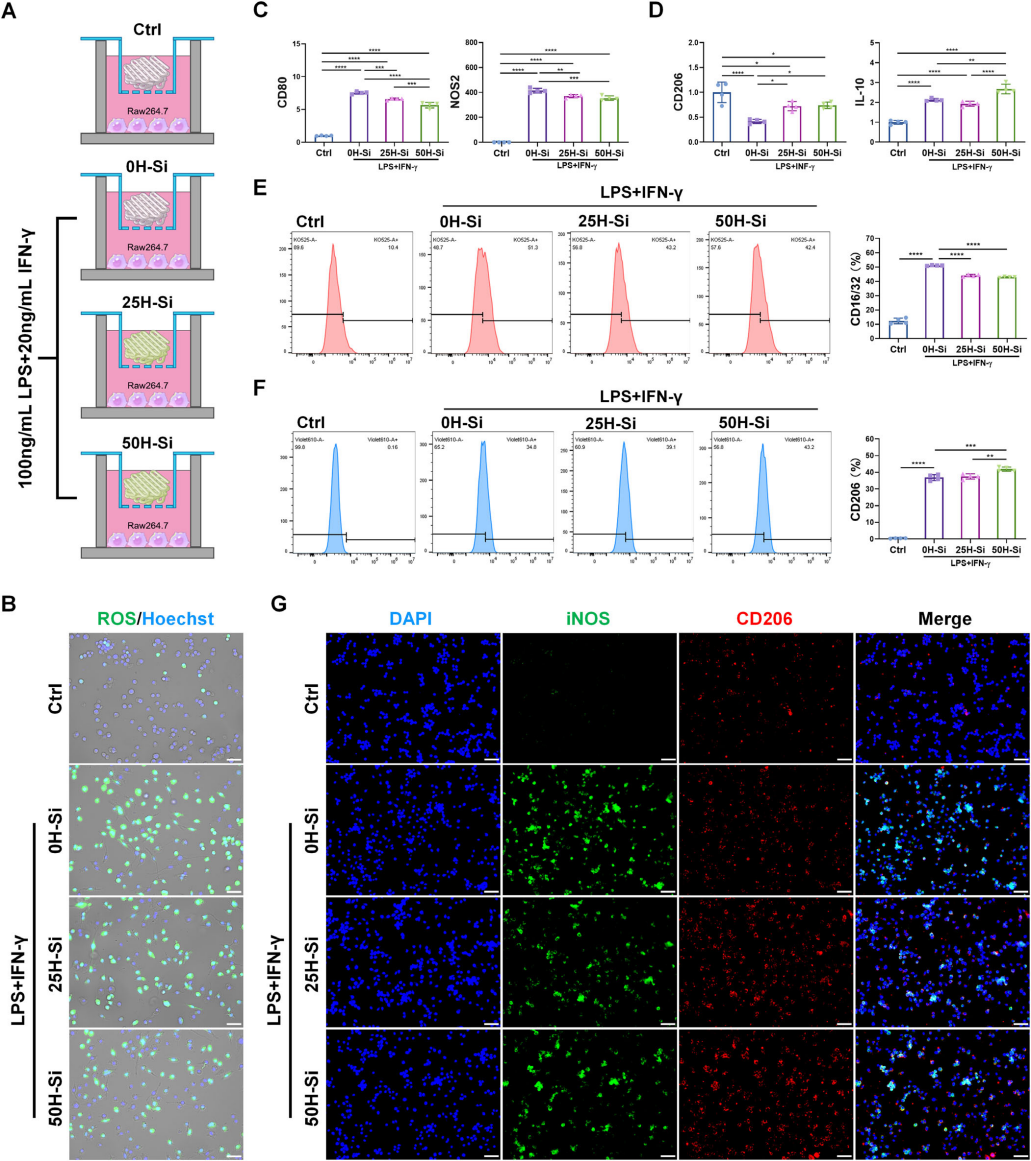
Immunoregulatory effect of tricalcium phosphate (TCP) and H‐Si TCP scaffold on macrophage polarization under inflammation. A) Schematic diagram of Raw264.7 macrophages incubated with TCP scaffold integrated with 0, 25, and 50 µg mL^−1^ H‐Si NSs with or without inflammatory stimulation. B) DCFH‐DA fluorescence staining (green fluorescence) to detect intracellular ROS production in Raw264.7macrophages under different treatments. The nuclei were stained with Hoechst 33342 (blue fluorescence). Bar = 50 µm. C,D) The expression level of M1 markers CD80 and NOS2, and M2 markers CD206 and IL‐10 in Raw264.7 macrophages under different treatments. *N* = 4, data were normalized to Ctrl group (set as 1). E,F) The flow cytometry of M1 marker CD16/32 and M2 marker CD206 expression in Raw264.7 macrophages under different treatments. *N* = 4. G) The immunofluorescent staining of iNOS (green fluorescence) and CD206 (red fluorescence) in Raw264.7 macrophages under different treatments. The nuclei were stained with 4′,6‐diamidino‐2‐phenylindole (DAPI) (blue fluorescence). Bar = 50 µm.^*^ *p* < 0.05; ^**^ *p* < 0.01; ^***^ *p* < 0.001; ^****^ *p* < 0.0001.

To assess the immunomodulatory effect of H‐Si TCP scaffolds on macrophage polarization under inflammation, Raw264.7 macrophages were incubated with different scaffolds together with LPS and IFN‐γ stimulation. The macrophages co‐cultured with TCP scaffold which received no LPS plus IFN‐γ treatment were set as control (Ctrl) (Figure 3A). As shown in Figure S4, 25H‐Si TCP, and 50H‐Si TCP scaffolds were able to upregulate both M1 and M2 macrophage polarization when inflammatory stimuli were absent. In particular, the fold changes of upregulated M2 macrophage polarization were much higher than that of M1 macrophages, indicating the potential immunomodulatory capacity of H‐Si TCP scaffold in tissue healing. While in the context of inflammation, H‐Si TCP could significantly reduce the overproduction of intracellular ROS in macrophages through the sustained release of H‐Si NSs from the scaffolds. This ROS‐scavenging effect was most prominent in 50H‐Si TCP group, with least green fluorescence observed among experiment groups (Figure 3B and Figure S5). The typical markers of macrophage differentiation were later carefully examined by qRT‐PCR and flow cytometry. As shown in Figure 3C,D, H‐Si TCP could not only significantly downregulate the expression level of typical M1 macrophage markers CD80 and nitricoxidesynthase‐2 (NOS2), but also greatly enhance typical M2 macrophage markers CD206 and interleukin‐10 (IL‐10) expression in macrophages, indicating a robust M2‐promotive effect on macrophage polarization under inflammation. Flow cytometry analysis also revealed the strong immunosuppression of H‐Si TCP on M1 polarization, in which the proportion of positive CD16/32 cells was significantly downregulated in 25H‐Si TCP and 50H‐Si TCP treatment (Figure 3E). In the meantime, H‐Si TCP could profoundly facilitate macrophage differentiation into M2 phenotype, as unraveled by upregulated proportion of positive CD206 cells (Figure 3F). The immunoregulatory effects of H‐Si TCP on macrophages polarization were further confirmed by immunofluorescence, as significantly less induced nitricoxidesynthase (iNOS) but stronger CD206 fluorescent staining was observed in both 25H‐Si TCP and 50H‐Si TCP group (Figure 3G). In conclusion, this evidence strongly illustrated the potent immunomodulatory effect of H‐Si TCP scaffold, with 50H‐Si TCP exhibiting the best M2‐promotive function. The H‐Si TCP scaffolds were enabled to reprogram macrophage polarization into anti‐inflammatory M2 phenotype and regulate the local immune microenvironment in defect sites, and thus could potentially maximize tissue regeneration outcomes in vivo.^[^ ^15^ ^]^

### 2.4 H‐Si TCP promoted the osteogenic differentiation of BMSCs in vitro

**FIGURE 4:**
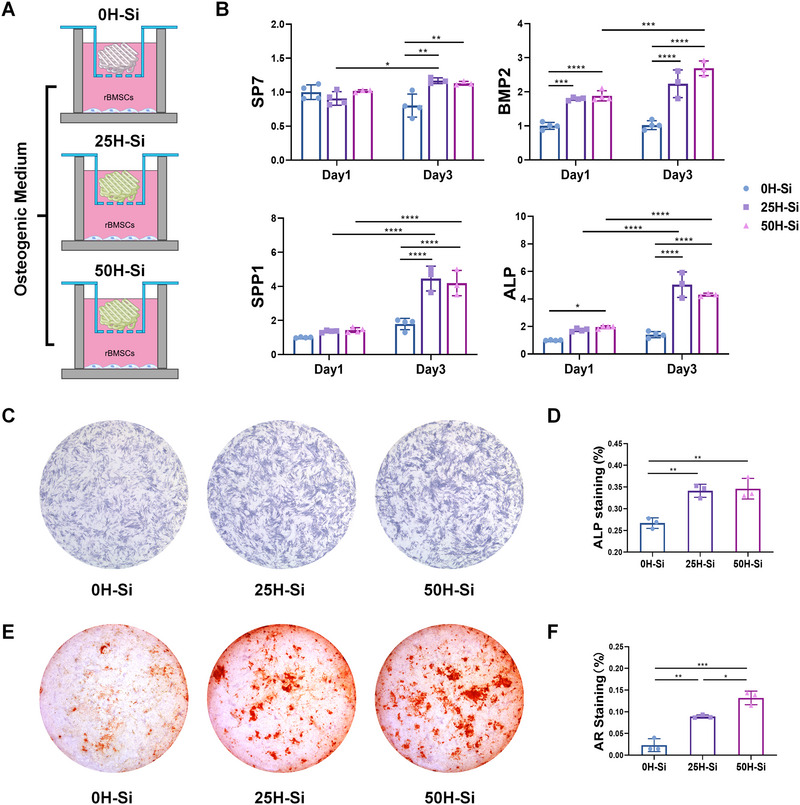
Effect of tricalcium phosphate (TCP) and H‐Si TCP scaffold on bone marrow mesenchymal stem cells (BMSCs) osteogenesis. A) Schematic diagram of BMSCs incubated with TCP scaffold integrated with 0, 25, and 50 µg mL^−1^ H‐Si NSs in osteogenic medium. B) The expression level of osteogenic markers SP7, bone morphogenetic protein‐2 (BMP2), SPP1 and alkaline phosphatase (ALP) in BMSCs at days 1 and 3. *N* = 3 or 4. Data were normalized to 0H‐Si at day 1 (set as 1). C) Alkaline phosphatase staining of BMSCs at day 7. D) Percentage of positive alkaline phosphatase staining area under different treatments. *N* = 3. E) Alizarin red staining of BMSCs at day 14. F) Percentage of positive Alizarin red staining area under different treatments. *N* = 3. ^*^ *p* < 0.05; ^**^ *p* < 0.01; ^***^ *p* < 0.001; ^****^ *p* < 0.0001. LPS, lipopolysaccharide; IFN‐γ, Interferon‐γ.

Mounting evidence has revealed that the incorporation of silicon could significantly improve the osteogenic capacity of the implanted biomaterials.^[^ ^16^ ^]^ Therefore, the osteo‐promotive effect of H‐Si TCP scaffold was evaluated in our study. BMSCs were co‐cultured with TCP, 25H‐Si TCP, and 50H‐Si TCP scaffolds in osteogenic medium (Figure 4A), and qRT‐PCR was conducted to assess the expression of typical osteogenic markers at days 1 and 3. All the data were normalized to TCP group at day 1. On day 1, the expression level of bone morphogenetic protein‐2 (BMP2) was significantly increased in 25H‐Si TCP and 50H‐Si TCP group, and alkaline phosphatase (ALP) expression was greatly enhanced in 50H‐Si TCP group. On day 3, the expression levels of BMP2, ALP, SPP1, and SP7 were all significantly upregulated in both 25H‐Si TCP and 50H‐Si TCP group compared with TCP group (Figure 4B). No significant difference was seen in RUNX2 and osteocalcin (OCN) expression at days 1 and 3 between different groups (Figure S6). Particularly, the fold changes of upregulated BMP2, ALP, SPP1 and SP7 expression in 25H‐Si TCP and 50H‐Si TCP groups at day 3 were significantly higher than the first day. However, no significant differences were observed in TCP group between days 1 and 3, indicating the initiation of osteogenesis was greatly promoted by H‐Si TCP scaffolds (Figure 4B). According to our qRT‐PCR results, 50H‐Si TCP played better role in bone formation than the other two groups in vitro.

The late osteogenic effect of H‐Si TCP scaffolds was further evaluated by alkaline phosphatase and alizarin red staining. In accordance with our qRT‐PCR results, BMSCs co‐cultured with H‐Si TCP scaffolds started to show stronger alkaline phosphatase staining than TCP group at day 3 (especially in 50H‐Si TCP) (Figure S7). This significance was even more obvious at day 7, with 25H‐Si TCP and 50H‐Si TCP group exhibiting prominently more robust alkaline phosphatase staining compared to the TCP group (Figure 4C). Alizarin Red staining also showed markedly denser calcium deposition in both H‐Si TCP scaffolds (especially in 50H‐Si TCP group) at day 14, while little to no positive staining was observed in TCP group (Figure 4E). The proportion of positive staining area was further quantified in alkaline phosphatase and alizarin red staining, and great significance was observed among three groups (Figure 4D,F).

Based on our results, H‐Si TCP scaffolds could substantially bring forward the initiation of osteogenesis and promote the osteogenic differentiation of BMSCs in vitro. This finding could attribute to the activation of key signaling pathways related to osteogenesis and the promotion of osteogenic genes expression by the sustained release of ionic silicon.^[^ ^17^ ^]^ Besides, silicon was engaged in active calcification in early stages of biomineralization and facilitated the crystallization and precipitation of hydroxyapatite during the later stages of calcification as well.^[^ ^18^ ^]^ As a result, our composite scaffold was very likely to further facilitate osteogenic process and potentially promote bone formation in vivo when integrated with H‐Si NSs.

### 2.5 H‐Si NSs significantly facilitate TCP scaffold degradation in vivo

**FIGURE 5:**
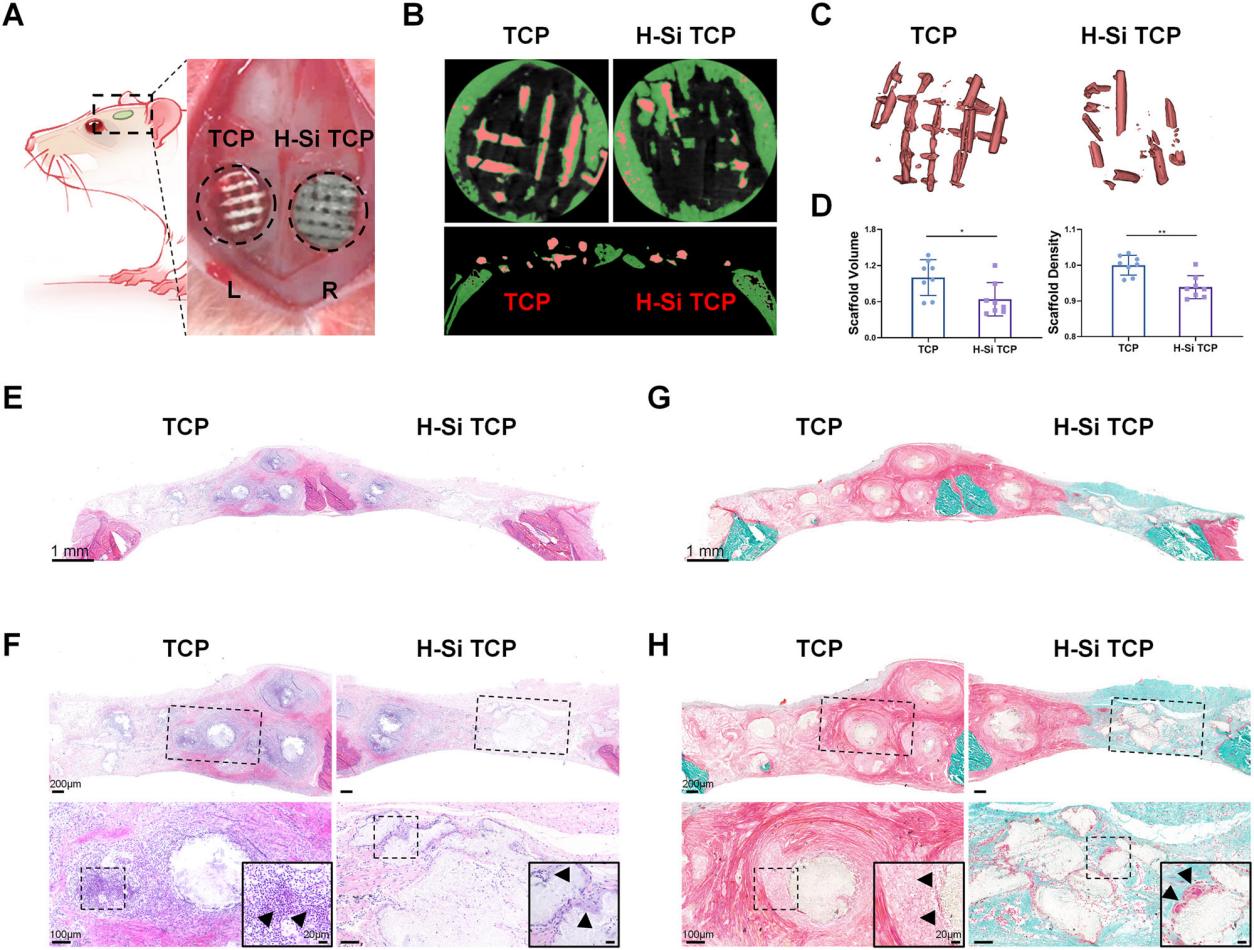
The biodegradability of tricalcium phosphate (TCP) and H‐Si TCP scaffold in cranial defects. A) Schematic diagram of scaffolds implantation in vivo. The SD rats were sacrificed after 8 weeks of implantation. B) The coronal (upper) and transaxial (lower) section of TCP and H‐Si TCP scaffold in cranial defects. The residual scaffold was labeled in pink. C) 3D reconstruction of residual TCP and H‐Si TCP scaffold in cranial defects after 8 weeks of in vivo implantation. D) Histomorphometric analysis of residual TCP and H‐Si TCP scaffold in cranial defects. *N* = 8, ^*^ *p* < 0.05, ^**^ *p* < 0.01. E,F) H&E staining of residual TCP and H‐Si TCP scaffold in cranial defects. The TCP scaffold was surrounded by large amounts of monocytes. G,H) Goldner trichrome staining of residual TCP and H‐Si TCP scaffold in cranial defects. The TCP scaffold was wrapped in thick fibrous tissue. The black arrowheads on TCP side indicated the infiltration of monocytes, and the black arrowheads on H‐Si TCP side indicated the formation of foreign body giant cells along the scaffold. BMP2, bone morphogenetic protein‐2; ALP, alkaline phosphatase.

Due to its superior osteogenic and immunoregulatory performance in vitro, 50H‐Si TCP scaffold (labeled as H‐Si TCP) was selected and utilized in the following in vivo experiments. A total of fifteen 6‐week‐old male Sprague‐Dawley rats (SD rats) were used to evaluate the performance of H‐Si TCP in vivo. Briefly, after two large cranial defects (5 mm in diameter) were created, TCP and H‐Si TCP were implanted for 8 weeks before SD rats were sacrificed (Figure 5A). The liver, heart, spleen, lung, kidney, and brain of SD rats were harvested to assess the systemic toxicity of implanted composites. Organs were fixed and underwent H&E staining for histology analysis, and blood samples were taken for blood routine examination. As Figure S8 shows, no obvious abnormality was seen in histology of these vital organs. In addition, blood routine indicators such as red blood cell count and white blood cell count all fell within the normal range (Figure S9). Based on these results, our composite implants did not exert significant systemic toxicity in vivo.

Generally, the implanted scaffolds are immunogenic and able to cause unwanted foreign body reaction in vivo, adversely impacting scaffold biodegradation and tissue regeneration.^[^ ^19^ ^]^ Therefore, the biodegradability and biocompatibility of TCP and H‐Si TCP were evaluated in vivo in the first place. As shown in macroscopic and micro‐CT view, H‐Si TCP was greatly degraded on the right side, while minimal degradation was observed in TCP on the left side (Figure S10). Typical images of coronal and transaxial sections in micro‐CT were presented in Figure 5B, where scaffold was labeled in pink while the mineralized bone tissue was represented in green. 3D reconstruction of residual scaffolds was further displayed in Figure 5C. Both results illustrated the considerably better biodegradability of H‐Si TCP in vivo. The degradation of both scaffolds was further quantified by histomorphometric analysis, and a significant reduction of the scaffold volume and density was observed in H‐Si TCP (Figure 5D).

To provide a deeper insight into the biodegradability of both implants, H&E and Goldner trichrome staining were performed to identify their histopathological differences in vivo (Figure 5E–H). Although both scaffolds displayed decent biocompatibility in vitro, their performance in vivo turned out to be an entirely different story. Mineralized materials including TCP could potentially arouse oxidative stress and excessive generation of ROS in vivo.^[^ ^20^ ^]^ Typically, ROS were identified as profibrotic metabolites, which could facilitate the synthesis and activation of various cytokines and growth factors involved in fibrosis and were positively correlated with fibrosis at a late stage.^[^ ^21^ ^]^ Consequently, it is not surprising to find that TCP was wrapped in thick fibrous tissue, isolated from the surrounding tissues. On account of the ROS‐scavenging property of H‐Si NSs, environmental oxidant levels could be efficiently reduced, and considerably less fibrous encapsulation was observed around the H‐Si TCP scaffolds in vivo. On the other hand, intensive immune events ensued after foreign biomaterials implantation.^[^ ^22^ ^]^ As shown in Figure 5E–H, most of the TCP scaffold was surrounded by a large quantity of monocytes, suggesting the persistence of chronic and dysregulated inflammatory reactions after implantation that ultimately led to the development of pathological fibrosis.^[^ ^23^ ^]^ On the contrary, relatively less monocyte infiltration and fibrotic encapsulation were captured around H‐Si TCP scaffold, indicating that the immune responses were carried out in a well‐orchestrated manner on H‐Si TCP side. This finding could largely attribute to the immunoregulatory effect of H‐Si NSs, which functionally modulate the local immune microenvironment in defect sites. Besides, the elimination of excessive ROS might contribute to the decrease of pro‐inflammatory M1 macrophages as well for healthy functional repairing.^[^ ^24^ ^]^ Particularly, foreign body giant cells, the multinucleated giant cells formed by the migration and aggregation of macrophages, were gathering around the implants to participate in the biodegradation of H‐Si TCP scaffolds (enlarged image in Figure 5F,H). These cells were able to synthesize many degradative products like hydrolytic enzymes, and actively engaged in breaking down of the foreign implants.^[^ ^25^ ^]^ As a result, it is unsurprising that H‐Si TCP displayed superior biodegradability in vivo. Besides, the H‐Si NSs released from the scaffold would undergo ROS‐responsive degradation in oxidative context (Figure 1L‐N), leaving insignificant impact on surrounding tissues. The connective tissue (stained in green) in defect area would be later replaced by bony tissue as it happened in other sections of the H‐Si TCP group. All these results suggested that the H‐Si NSs integrated onto 3D printed TCP scaffolds were actively engaged in regulating immune microenvironment and eliminating detrimental oxidants in defect region and thus greatly facilitated composite scaffold biodegradation and integration with surrounding tissues in vivo.

### 2.6 H‐Si TCP greatly expedites osteogenic process in vivo

**FIGURE 6:**
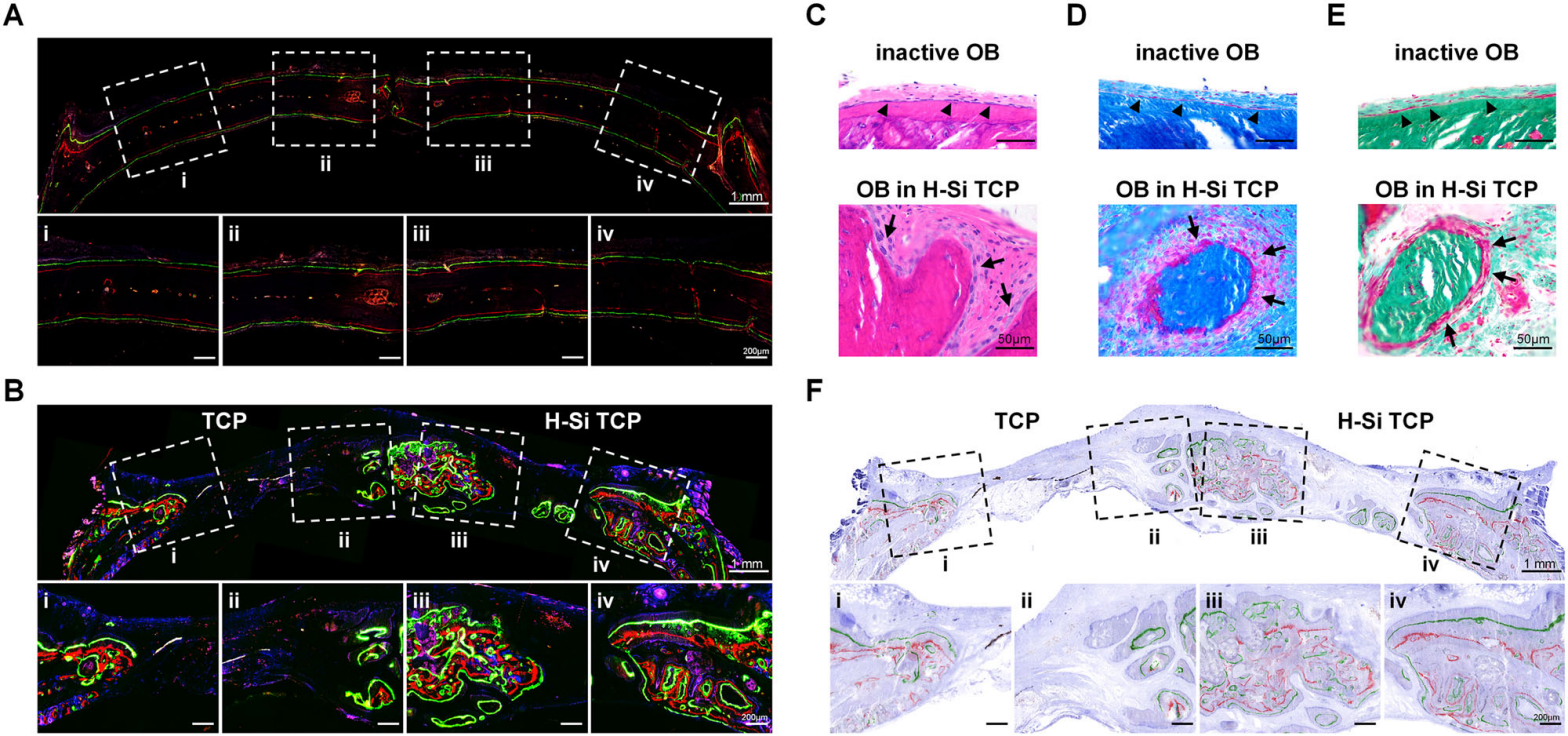
The osteo‐inducive capacity of tricalcium phosphate (TCP) and H‐Si TCP scaffolds. A,B) Tetracycline hydrochloride (blue), alizarin red (red), and calcein (green) were injected subcutaneously into rats with A) uninjured cranium or B) cranial defects at weeks 2, 4, and 6. Different colors of fluorescence represented newborn bone tissue formation at different periods. C) H&E staining, D) Masson trichrome staining, and, E) Goldner trichrome staining of inactive osteoblasts (flat, black arrowheads) in uninjured cranium and activated osteoblasts (cubic, black arrows) in defective cranium implanted with H‐Si TCP. F) The fluorescent labeling of osteoblastic trajectory in cranial defects was well‐located within the osseous tissue in the bright field. OB, osteoblasts.

Due to its great role in osteogenesis, the performance of H‐Si TCP in vivo is worth expecting. On one hand, H‐Si TCP could significantly promote osteogenic genes expression and bring forward the initiation of osteogenic activity. On the other hand, H‐Si TCP was able to upregulate M2 polarization and facilitate macrophages functional conversion into tissue‐repairing mode. Although the correlations between M2 macrophages and osteogenesis were not illustrated in our study, the beneficial effects of M2 macrophages on osteogenic activity and bone formation have been fully investigated and identified in numerous previous studies.^[^ ^26^ ^]^ Therefore, H‐Si TCP was supposed to potentially facilitate osteoblastic differentiation and activation, and promote new bone formation in vivo. Confocal laser scanning microscopy (CLSM) shed light on osteogenic activity of osteoblasts and newborn bone formation in cranial defects. Briefly, tetracycline hydrochloride (Tet‐HCl, blue), Alizarin red (red), and Calcein (green) were injected into rats sequentially as described in Experimental Section. Different colors represented the dynamic new bone formation at different timepoints. To be specific, the blue fluorescence represented the early osteogenesis on week 2, the red fluorescence indicated the ossification during week 4, while the green fluorescence reflected bone formation in the last 3 weeks. In uninjured cranium, the osteogenic activity was generally located along the periosteum, with almost inactive ossification ongoing within cranium (Figure 6A and Figure S11A). When injury occurred, however, it could arouse dynamic osteogenic activity. Highly active newborn bone tissue, which was indicated by different colors of fluorescence (Figure 6B and Figure S11B), was observed around the defect side, crawling along the pores of both scaffolds. Significantly, newborn osseous tissue was more actively accumulated along H‐Si TCP scaffold as manifested by the fluorescent labeling of all three colors, indicating better osteogenic performance and more new bone formation on the experiment group during the past 8 weeks. Particularly, the blue fluorescence, which labeled the early bone formation at week 2, was more accumulated on H‐Si TCP side (Figure S11B,C), indicating an earlier start of osteogenesis in cranial defects.

Histology provided a deeper perspective into the osteoblastic behavior in defect region. On the site of uninjured cranium, inactive osteogenesis was ongoing subperiosteally. The dormant osteoblasts were rested in flat shape, lying against the periosteal tissue (black arrowheads, Figure 6C–E). This histomorphology was consistent with CLSM results, in which only a few of linear fluorescent strips were observed along the periosteum. When injury occurred, quantities of BMSCs differentiated into functional osteoblasts, and were recruited and activated in defect region following the degradation of H‐Si TCP scaffold. As shown in Figure 6C–E, a large number of activated osteoblasts were observed in defective cranium on H‐Si TCP side. In particular, the osteoblasts turned into cubic morphology (black arrows), actively engaged in new bone formation. As the result, it came as no surprise to observe intensive fluorescence in defect region where H‐Si TCP was implanted. On the side of TCP, however, the scaffold was scarcely degraded and gathered around by a large number of inflammatory cells (Figure S12). Consequently, very few of fluorescent strips were found in defect region on TCP side.

Since the fluorescence was closely related to the osteogenic trajectory of activated osteoblasts, the fluorescent images were further merged with images in bright field. As shown in Figure 6F, the fluorescent tracks of activated osteoblasts were well‐located within the osseous tissue in the bright field, precisely indicating osteogenic process in defect regions. In addition, compared with TCP alone, considerably more osteoblasts were activated on H‐Si TCP side to participate in bone regeneration, and thus more osseous tissue was present around the H‐Si TCP. Given the above, H‐Si TCP implant could efficiently facilitate BMSCs recruitment, promote osteoblastic differentiation as well as activation, and expedite the new bone formation in vivo.

### 2.7 H‐Si TCP significantly promoted bone remodeling in vivo

**FIGURE 7:**
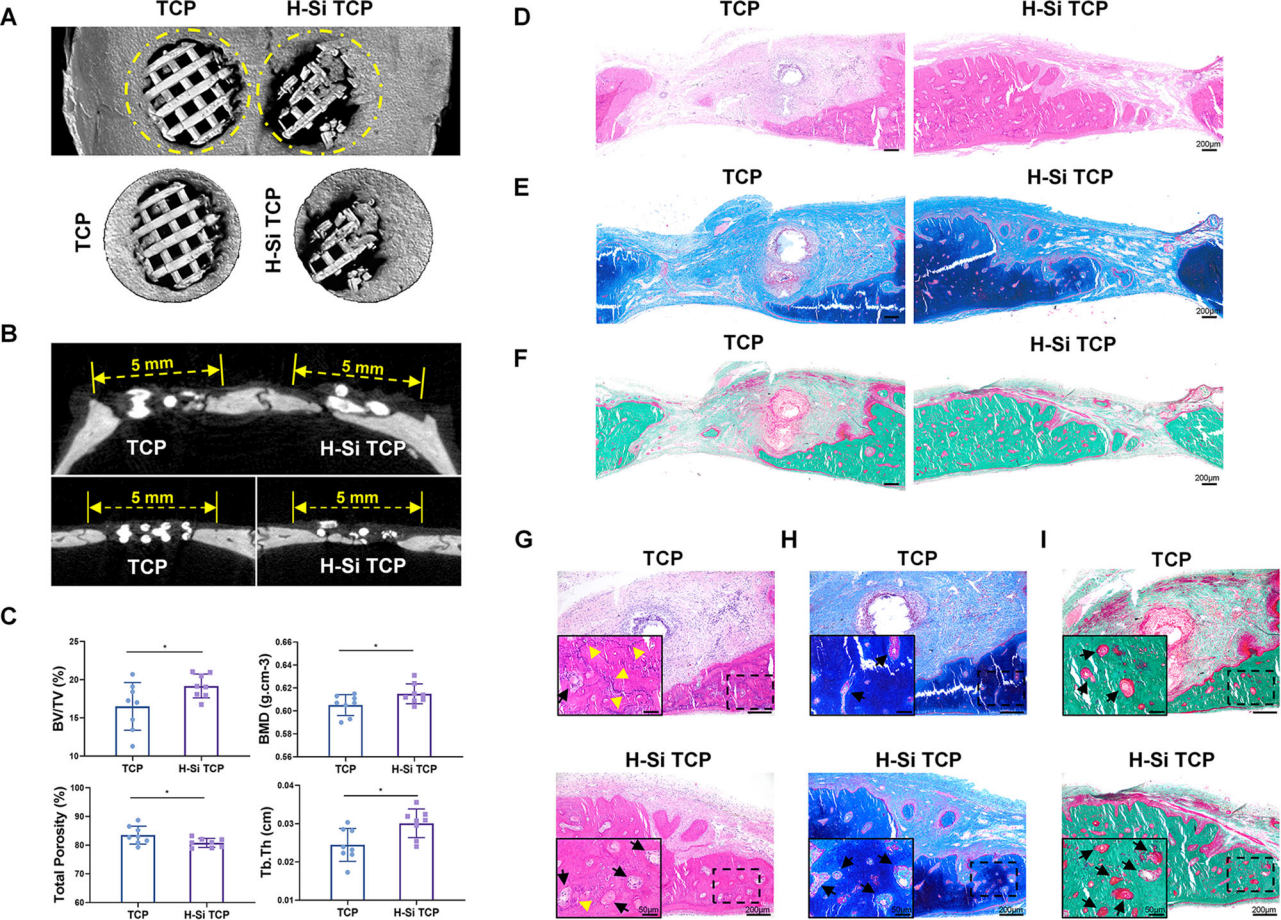
The material‐guided biomineralization of tricalcium phosphate (TCP) and H‐Si TCP scaffolds. A) Typical 3D reconstruction image of cranial defect implanted with TCP and H‐Si TCP scaffolds. Yellow circles marked the original defect area. B) The typical transaxial (upper) and sagittal (lower) sections of cranial defect implanted with TCP and H‐Si TCP scaffolds. Yellow dotted lines indicated the original defect area. C) Histomorphometric analysis of bone regeneration in cranial defect implanted with TCP and H‐Si TCP scaffolds. *N* = 8, ^*^ *p* < 0.05. D) H&E staining, E) Masson trichrome staining, and F) Goldner trichrome staining of cranial defects implanted with TCP and H‐Si TCP scaffolds. G) H&E staining, H) Masson trichrome staining, and, I) Goldner trichrome staining of newborn osseous tissue on TCP and H‐Si TCP side. The black arrows indicated the newly formed osteoid and the yellow arrowheads in (G) indicated the cement lines. OB, osteoblasts.

As we discussed before, calcium and phosphorus are essential ingredients for mineralization process in vivo. TCP could serve as reservoir for calcium and phosphorus but require adequate degradation. Due to superior biodegradability of H‐Si TCP in vivo, its degradation could provide sufficient products for bone mineralization. Besides, silicon was deemed as an important component in bone tissue which was vital for bone formation and maintenance.^[^ ^27^ ^]^ Thus, the released calcium, phosphate radical, and silicon following H‐Si TCP degradation could collectively promote the biomineralization and regenerative process in defect region. As the macroscopic images shown in Figures S10A and S13A, much better bone repair of cranial defects was seen on the right side where H‐Si TCP scaffolds were implanted. The cranial defects were then 3D reconstructed and analyzed through micro‐CT scanning. The yellow signs marked the original defective area in cranium. As shown in Figure 7A, more mineralized tissue was present on the side of H‐Si TCP. When looking from the coronal and sagittal plane, defect implanted with H‐Si TCP was better healed, with the gap greatly narrowed from both ends (Figure 7B). The bone defect regeneration was further evaluated by quantitative analysis of fundamental histomorphometric parameters (Figure 7C). The result showed that the bone volume/total volume (BV/TV), bone mineral density (BMD), and trabecular thickness (Tb.Th) of the experiment group were greatly enhanced while the total porosity was correspondently reduced.

These results consistently illustrated the significantly improved osteo‐inducive capacity of H‐Si TCP. Surprisingly, both scaffolds were completely degraded in seven SD rats (Figure S13A), possibly because of better surgical techniques (making a better adhesion of the material to the defect side) and postoperative recovery. Micro‐CT analysis also showed the same results that the H‐Si TCP side healed better than the TCP side (Figure S13A,C), with less gap across the defect regions in coronal and sagittal section (Figure S13B). In addition, histomorphometric parameters of micro‐CT analysis (Figure S13D) also exhibited similar results, indicating better bone mineralization of H‐Si TCP. However, no significant difference of bone mineral density (BMD) was observed between H‐Si TCP and TCP group, probably because of the low density of unmineralized newborn osseous tissue.

Histological staining of hard tissue slices further confirmed the superior bone regenerative capacity of H‐Si TCP scaffolds. As shown in Figure S14 and Figure 7D–F, the defect on H‐Si TCP side was greatly narrowed, replaced by massive newborn osseous tissue generated from both ends. In contrast, most part of the defect was filled with connective tissue around the TCP scaffolds. Higher magnification was further acquired to illustrate the histopathological differences between two groups. On the edges of regenerated defects, plenty of osteoid (indicated by black arrows in Figure 7G–I), which was the matrix of type I collagen and glycosaminoglycans produced by osteoblasts around them, were observed on H‐Si TCP side, revealing the highly dynamic osteogenic activity. Eventually, osteoid would become mineralized with calcium hydroxyapatite to produce bone, with mature osteocytes trapped within them.^[^ ^28^ ^]^ In contrast, considerably less osteoid were seen on the other side, indicating compromised new bone formation of the TCP scaffolds. It is worth noting that on the side of TCP, new bone tissue was demarcated by thick irregular cement lines (indicated as the wavy blue lines in H&E staining, pointed by yellow arrowheads in Figure 7G). These basophilic lines implied the boundary between calcified and non‐calcified areas during the bone formation process, which was almost invisible in mature osseous tissue.^[^ ^29^ ^]^ Cement lines exhibit a state of disordered growth when the physiologic dynamic balance of calcification is destroyed.^[^ ^30^ ^]^ As shown in Figure 7G, disorganized cement lines appeared in the TCP group, probably due to the insufficient supply of calcium and phosphorus. On the contrary, sparse cement lines were found in the newborn bone tissue around H‐Si TCP scaffolds (Figure 7G), suggesting that the disturbed process of bone mineralization and regeneration happened in TCP was greatly smoothed out when H‐Si NSs were integrated onto the scaffold.

In short, due to the superior biocompatibility and biodegradability of H‐Si TCP in vivo, the dissociation of H‐Si TCP scaffold would provide ample ingredients (mainly calcium and phosphate) for biomineralization and ossification, and give way to new bone formation in situ. Besides, the silicon released from H‐Si NSs and M2‐promotive effect of H‐Si TCP could collectively promote the osteogenic differentiation of BMSCs. By contrast, on the side of TCP, the disturbed inflammatory responses and thick fibrous encapsulation greatly blocked the scaffold biodegradation as well as bone mineralization and thus resulted in unfavorable bone regeneration and integration. Hence, it hardly comes as a surprise to witness newborn bone on TCP side of less amount and inferior quality. Of note, neovascularization was also observed along the H‐Si TCP scaffolds (Figure S15), probably because of the pro‐angiogenic property of silicon, as indicated in former study.^[^ ^31^ ^]^

## 3 CONCLUSIONS

In summary, our study developed a novel 2D H‐Si NSs integrated with 3D‐printed β‐TCP as multifunctional bone‐mimetic H‐Si TCP scaffolds, which possessed smart ROS‐regulatory effect, robust immunoregulatory property, potent osteo‐inducive capacity as well as excellent biocompatibility and biodegradability in vivo. Of note, osteoimmunomodulation was proposed and proved as the key reason for the superior biodegradation and bone regeneration in H‐Si TCP scaffolds. This process was carried out in a spatiotemporal pattern that was fully elaborated in Scheme 1. As we know, the implantation of foreign biomaterials would induce severe foreign body responses, and elicit increased oxidative stress and inflammatory reactions in defect region, just as we observed in TCP scaffold. However, H‐Si TCP implants would profoundly improve the local immune microenvironment in vivo. On one hand, H‐Si NSs released from the composite scaffold could quench the excessive oxidants in situ. On the other hand, H‐Si NSs could enter macrophages via endocytosis and downregulate the intracellular ROS level, polarizing the macrophages into M2 phenotype. The functional conversion from pro‐inflammatory into anti‐inflammatory microenvironment greatly brought down the undesired immunogenic responses and orchestrated scaffold degradation and bone regenerative process in defect region. Then efficient osteogenesis and mineralization took place following the biodegradation of the composite scaffold. BMSCs were recruited and differentiation into functional osteoblasts, and actively engaged in new bone formation. The degradation of H‐Si TCP provided adequate inorganic ingredients for biomineralization and enough space for bone ingrowth, and thus ensured the favorable and functional bone regeneration in vivo. In short, the hydrogenated silicon nanosheets functionalized scaffold developed in our study enables immuno‐bone remodeling, which plays a critical role in the spatiotemporal orchestration of scaffold degradation and bone regeneration in vivo. Therefore, H‐Si TCP could be a promising substitute to fix large bone defects in the near future.

## 4 EXPERIMENTAL SECTION

Experimental details are provided in the Supporting Information.

## CONFLICT OF INTEREST STATEMENT

Han Lin is a member of the *Exploration* editorial board. The authors declare no conflict of interest.

## Supporting information

Supporting Information

## Data Availability

All data needed to evaluate the conclusions in the paper are present in the paper and/or in the Supporting Information. Additional data related to this paper may be requested from the authors.
