# Supplementary material for: Hydrogenated silicene nanosheet functionalized scaffold enables immuno‐bone remodeling
Source: Exploration (Beijing). 2023 May 28;3(4):20220149. doi: 10.1002/EXP.20220149 (PMC10624372; doi:10.1002/EXP.20220149)
Supplement: Supplementary file 1 — Supporting Information [file EXP2-3-20220149-s001.docx]

Supporting Information

**Hydrogenated Silicene Nanosheet Functionalized Scaffold Enables Immuno-Bone Remodeling**

Zixuan Lin^1^, Zhixin Chen^2^, Yiwei Chen^1^, Nan Yang^1^, Jianlin Shi^2,3^, Zhongmin Tang^4^, Changqing Zhang^1*^, Han Lin^2,3*^, and Junhui Yin^1*^

1. Institute of Microsurgery on Extremities, Department of Orthopaedic Surgery, Shanghai Sixth People's Hospital Affiliated to Shanghai Jiao Tong University School of Medicine, Shanghai 200233, P. R. China.

2. State Key Laboratory of High Performance Ceramics and Superfine Microstructure, Shanghai Institute of Ceramics Chinese Academy of Sciences, Shanghai 200050, P. R. China.

3. Shanghai Tenth People's Hospital, Shanghai Frontiers Science Center of Nanocatalytic Medicine, School of Medicine, Tongji University, Shanghai 200331, P. R. China.

4. Departments of Radiology and Medical Physics, University of Wisconsin-Madison, Madison, Wisconsin, USA.

*Corresponding author: zhangcq@sjtu.edu.cn; linhan@mail.sic.ac.cn; yinjunhui1429@126.com

Zixuan Lin and Zhixin Chen have contributed equally to this work.

**Experimental Section**

**Synthesis of H-Si TCP scaffolds**

The 1000 mg Zintl-phase calcium silicide (CaSi_2_, tech-95, 95%, powder, Gelest) was immersed and stirred in 100 mL of precooled hydrochloric acid (HCl, 36%, Caledon Laboratory Chemicals) at ∼ -20 °C under the Ar flow protection for one week. The as-prepared sample was performed by centrifugation and washing with anhydrous ethanol for two times. Such multilayered H-Si NSs were suspended in anhydrous ethanol and further sonicated for 12 h, to obtain the few-layered H-Si NSs with smaller lateral size. The beta-tricalcium phosphate (β-TCP) scaffolds were obtained according to our previous reports. The scaffolds were soaked in H-Si NSs aqueous solution (25°C, 30 min) and then put in the drier (37°C, 2 h). The H-Si TCP scaffolds were obtained after the above steps (repeated three times).

**Characterization**

Transmission electron microscopy (TEM) images, and energy dispersive X-ray spectroscopy (EDS) were performed on JEM-2100F transmission electron microscope operated at 200 kV and spherical aberration correction equipped JEOL ARM-300F was operated at 300 kV. UV-vis-NIR absorbance analysis was conducted on UV-3600 Shimadzu UV-vis-NIR spectrometer. The quantitative elemental analysis was conducted on inductively coupled plasma-optical emission spectrometry (ICP-OES, Agilent 700 Series, Agilent Technologies, US).

**Measurement of ABTS^+•^, ∙OH, O_2_^•−^ and H_2_O_2_ scavenging**

The total free radicals scavenging ability of H-Si NSs was determined by the Total Antioxidant Capacity Assay Kit with a Rapid ABTS method (T-AOC Assay Kit) and here was performed by the color change of ABTS (2,2'-azino-bis(3-ethylbenzthiazoline-6-sulfonic acid) as the developer. The superoxide dismutase (SOD) assay kit was used to determine the superoxide anion (O_2_^•−^) scavenging ability of H-Si NSs. The scavenging activity of H-Si NSs against hydroxyl radical (∙OH) was determined by the effective removal of ∙OH originated from Fenton reaction. FeSO_4_ (1 mM) and H_2_O_2_ (2 mM) were mixed in sodium acetate buffer, followed by the addition of H-Si NSs dispersion. After 5 minutes of reaction, 3,3’,5,5’-Tetramethylbenzidine (TMB) was used to test the absorbance at 650 nm. Ammonium molybdate can react with H_2_O_2_ to develop color for quantification. H_2_O_2_ reacted with H-Si NSs for 10 minutes in advance, and then ammonium molybdate color was added to test the absorbance of this system at 405 nm. All tests were performed in quintuplicate (n = 5).

**CCK-8 assay**

Rat bone-marrow mesenchymal stem cells (BMSCs) were seeded in 96-well plates at a density of 1.0×10^4^ cells per well and cultured in growth medium (GM, α-MEM (Gibco, Grand Island, NY) supplemented with 10% fetal bovine serum (Gibco, Grand Island, NY) and 1% penicillin/streptomycin (Gibco, Grand Island, NY)). To assess the cytotoxicity of H-Si TCP scaffolds, different concentrations of H-Si NSs (0, 25, 50, 100, 250 and 500 µg mL^-1^) integrated with β-TCP scaffolds were added to GM respectively and incubated with BMSCs for 24 hours. The viability of BMSCs was tested by Cell Counting Kit-8 (CCK-8) assay following the manufacturer’s protocol. As the result shown, the scaffolds started to show significant cytotoxicity when the concentration of H-Si NSs reached 100 µg mL^-1^ or more. To further evaluate the long-time cytotoxicity of TCP, 25H-Si TCP and 50H-Si TCP scaffolds, BMSCs were incubated with these scaffolds respectively for 7 days, and CCK-8 assay was conducted on day 1, 3, 5 and 7.

**Live/dead and cytoskeleton fluorescent staining**

The scaffolds were first sterilized by ultraviolet light overnight, and then a high concentration of cell suspension was added onto the surface of TCP, 25H-Si TCP and 50H-Si TCP scaffolds. GM was later gently added to the culture after the cells were preliminarily adhered. To assess the viability of BMSCs on scaffolds, the samples were harvested and co-stained with calcein acetoxymethyl ester (calcein-AM, green fluorescence) and propidium iodide (PI, red fluorescence) at day 1. To examine the adhesion and proliferation of BMSCs on scaffolds, the nuclei of BMSCs were stained with 4′,6‐diamidino‐2‐phenylindole (DAPI, blue) and the cytoskeletons of BMSCs were stained by rhodamine phalloidin (red) at day 1 and 7. All images were taken by an Olympus IX81 microscope with a UC90 camera.

**Raw264.7 macrophage culture and polarization**

Raw264.7 macrophages were seeded in 12-well plates at a density of 5.0×10^5^ per well and cultured in high-glucose Dulbecco's modified Eagle's medium (DMEM) supplemented with 2% FBS. Raw264.7macrophages was then incubated with TCP, 25H-Si TCP and 50H-Si TCP scaffolds for 12 hours with or without 100 µg mL^-1^ lipopolysaccharide (LPS) plus 20 µg mL^-1^ Interferon-γ (IFN-γ) (Peprotech, Rocky Hill, NJ) stimulation, which was added to simulate inflammation as well as induce macrophage polarization.

**BMSCs osteogenic differentiation**

BMSCs were seeded in 6-well and 48-well plates at a density of 2.0×10^5^ and 2.0×10^4^ per well respectively. Upon reaching 70%-80% confluency, the BMSCs were co-cultured with TCP, 25H-Si TCP and 50H-Si TCP scaffolds in osteogenic medium (high-glucose DMEM supplemented with 10% FBS, 1% penicillin/streptomycin, 0.1 µM dexamethasone, 10 mM β-glycerophosphate (Sigma-Aldrich, St. Louis, MO, USA), and 50 µM L-ascorbic acid (Sigma)) to analyze the osteogenic capacity of H-Si TCP scaffolds.

**Quantitative real-time polymerase chain reaction (qRT-PCR)**

Total RNA of Raw264.7 macrophages and BMSCs were harvested using TRIzol reagent (Invitrogen). PrimeScript RT reagent kit (Takara, Shiga, Japan) was used for mRNA reverse transcription into complementary DNA, and ABI 7900 was used for quantitative analysis of the reverse transcription reaction. The relative gene expression of osteogenic makers such as bone morphogenic protein 2 (*BMP2*), alkaline phosphatase (*ALP*), secreted Phosphoprotein 1 (*SPP1*), *SP7* and M1/M2 macrophage markers *CD80*, nitricoxidesynthase-2 (*NOS2*), *CD206*, interleukin-10 (*IL-10*) were analyzed by the 2^−ΔΔ^Ct method. Glyceraldehyde-3-phosphate dehydrogenase (*GAPDH*) was used as housekeeping gene control.

**Reactive Oxygen Species generation evaluation**

DCFH-DA fluorescence staining was performed according to assay kit manufacturer’s instructions after Raw264.7 macrophages polarization. Hoechst 33342 was used to stain nuclei. All images were taken by an Olympus IX81 microscope with a UC90 camera.

**Flow cytometry**

After co-culture, Raw264.7 macrophages were trypsinized, washed and incubated with the Brilliant Violet 510 anti-mouse CD16/32 and Brilliant Violet 605 anti-mouse CD206 (BioLegend, SD, CA) in 4℃ for 30 minutes. Cells were then washed again and analyzed by flow cytometry (BD FACS AriaTM II cell sorter; BD Biosciences) to assess their surface epitopes expression.

**Immunofluorescent staining**

The Raw264.7 macrophages were first fixed and blocked in 10% horse serum (Vector Labs, Burlingame, CA, USA) in PBS for 1 hour. Then, the macrophages were incubated with primary antibody against mouse inducible nitric oxide synthase (iNOS) and CD206 (R&D Systems, Minneapolis, MN) overnight at 4°C. Fluorescein isothiocyanate (FITC)-conjugated secondary antibody was used to indicate the positive staining, and nuclei were stained by DAPI. All images were taken by an Olympus IX81 microscope with a UC90 camera.

**Alkaline phosphatase and Alizarin red staining**

To evaluate alkaline phosphatase activity, BMSCs were fixed with 4% paraformaldehyde and stained with alkaline phosphatase assay kit (Sigma) on day 3 and 7 following the manufacturer’s protocol. To assess the extracellular calcium deposition, BMSCs were fixed and stained in Alizarin Red S (2% aqueous, Sigma) solution for 30 min on day 14. The images were taken by LEICA PT2500E. The proportion of positive staining area in alkaline phosphatase and Alizarin Red staining was quantified and analyzed by ImageJ software.

**Surgical models of calvarial defect and scaffolds implantation**

All animal experiments were approved by the Research Ethics Committee of Shanghai Jiao Tong University Affiliated Sixth People's Hospital. As 50H-Si TCP is more effective for M2 polarization and BMSCs osteogenic differentiation, β-TCP scaffolds were integrated with 50 µg mL^-1^ H-Si NSs for *in vivo* study. A total of fifteen male Sprague–Dawley (SD) rats were used for surgical models of large calvarial defect in order to comprehensively inspect the bone healing effect of β-TCP scaffold (TCP) and β-TCP scaffold integrated with 50 µg mL^-1^ H-Si TCP *in vivo*. In detail, after anesthesia and sterilization, the skin was incised to expose the calvarial sagittal suture. Then, two 5-mm-diameter defects were made in the frontal parietal bone using a slow speed electric trephine (Nouvag AG, Goldach, Switzerland). The defects were filled with TCP scaffolds on the left side and H-Si TCP scaffolds on the right side. Finally, the periosteum and skin were sutured separately to close the incision (**Figure 5A**). Tetracycline hydrochloride, Alizarin red and Calcein (Sigma) were injected subcutaneously at week 2, 4 and 6 after *in vivo* implantation of the scaffolds. At the end of 8 weeks, the rats were executed. The calvarial specimens and organs including heart, liver, lung, kidney, spleen and brain were obtained and fixed in 10% buffered formalin phosphate. The blood samples were taken for blood routine examination (two blood samples suffered severe hemolysis).

**Micro-CT assay**

The harvested calvarial samples were scanned with a micro-CT scanner (Skyscan, Kontich, Belgium). The specimens were evaluated at a resolution of 18 μm per voxel following the established protocol. The CT images of calvarial samples were 3D reconstructed and analyzed. Histomorphometric parameters including bone mineral density (BMD), bone volume ratio (BV/TV), total porosity, trabecular thickness (Tb.Th) and trabecular number (Tb.N) of the newborn osseous tissue as well as scaffold volume and density were calculated using the auxiliary software of the mCT-80 system.

**Histology Staining**

The samples were dehydrated and embedded in hard plastic resin and carefully sectioned to examine the systematic toxicity of implants as well as newborn bone generation and scaffold degradation in cranial defects. Hematoxylin and Eosin (H&E), Masson trichrome and Goldner trichrome staining were then performed. Briefly, for H&E staining, the sections were stained in hematoxylin and eosin solution for 10 min respectively. For Masson trichrome staining, the sections were stained in hematoxylin solution for 5 minutes, ponceau acid fuchsin solution for 10 minutes, and then in phosphomolybdic acid-orange G solution and aniline blue solution for 5 minutes separately. For Goldner trichrome staining, sections were kept in Weigert’s hematoxylin solution and ponceau acid fuchsin solution for 30 min, and then sequentially in phosphomolybdic acid-orange G solution for 5 minutes and light green solution for 15 minutes. The photomicrographs were taken by LEICA DM 4000 microscope.

**Statistical Analysis**

Statistical analyses were performed with one-way and two-way analysis of variance (ANOVA) or two-tailed t-tests using GraphPad Prism 8 (GraphPad Software, San Diego, CA, USA). The data were presented as mean ± standard deviation and p < 0.05 value was considered statistically significant.


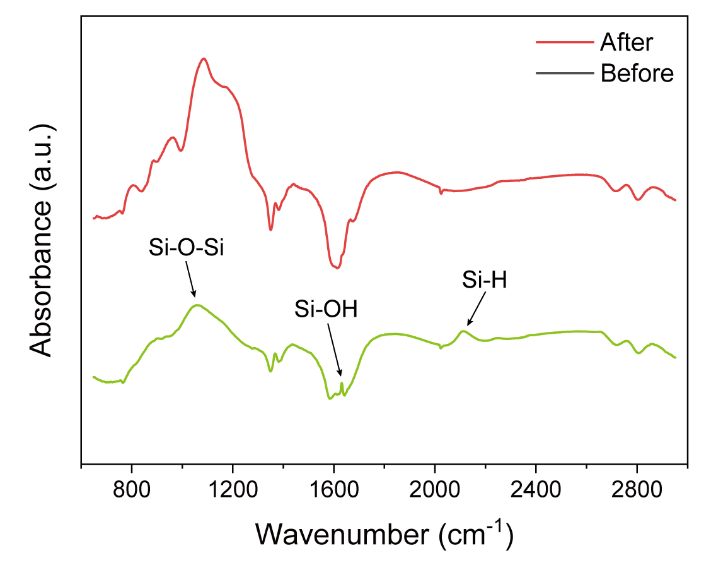


**Figure S1.** The infrared spectrum of the H-Si NSs before and after the scavenging reaction.

**Figure S2.** The ratio of live and dead cells on TCP scaffold integrated with different concentrations of H-Si NSs at day 1. The data were normalized to 0H-Si (set as 1). *N* = 3. No significant difference was seen between different groups.

**
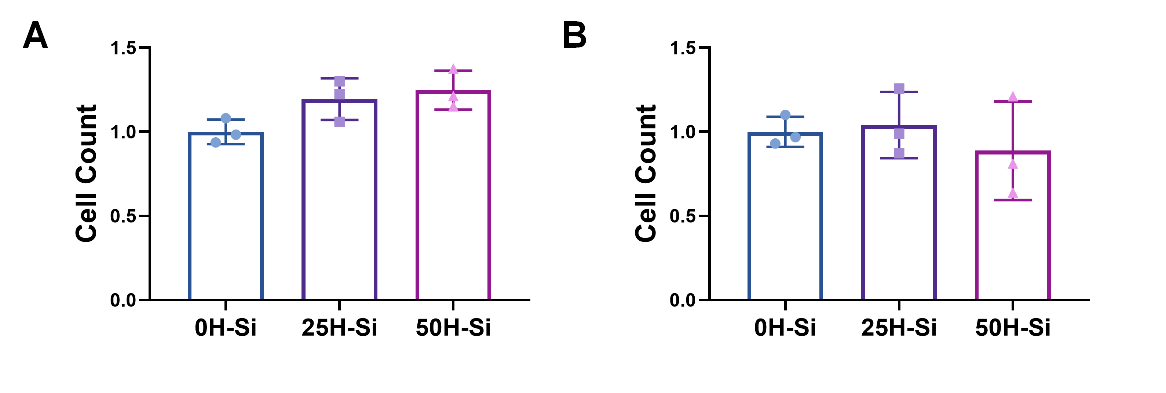
**

**Figure S3.** The cell number of BMSCs on TCP scaffold integrated with different concentrations of H-Si NSs at (**A**) day 1 and (**B**) day 7. The data were normalized to 0H-Si (set as 1). *N*=3. No significant difference was seen between different groups at day 1 and 7.


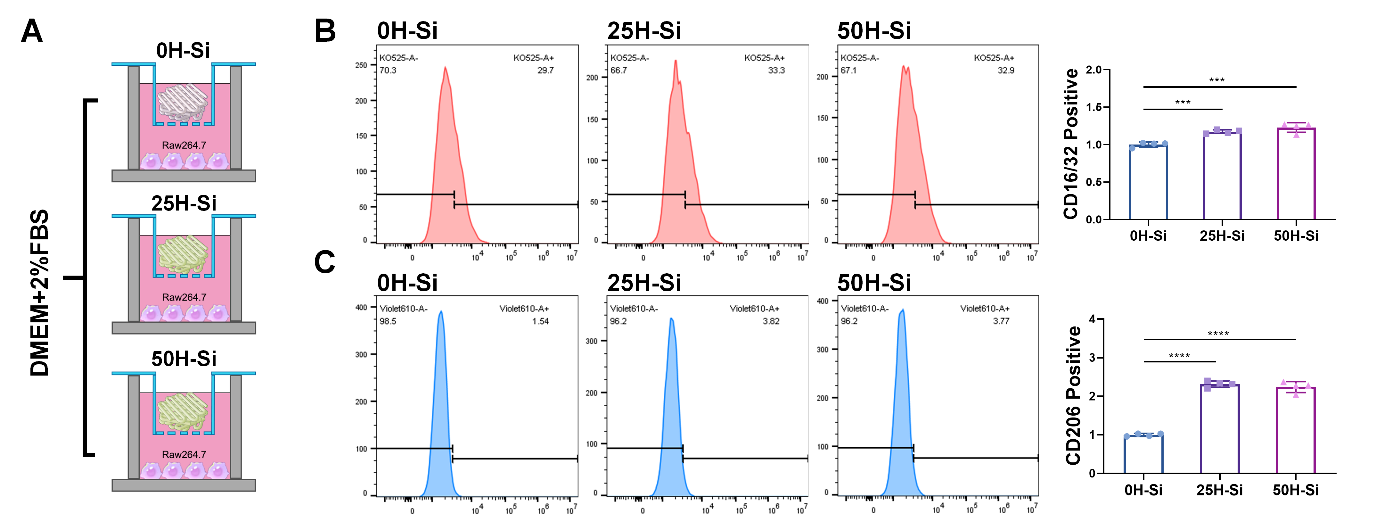
 **Figure S4.** The immunoregulatory effect of TCP and H-Si TCP on macrophages polarization without inflammatory stimulation. (**A**) Schematic diagram of Raw 264.7 macrophages incubated with TCP scaffold integrated with 0, 25 and 50 μg mL-1 H-Si for 12 h. (**B, C**) The flow cytometry of M1 marker CD16/32 and M2 marker CD206 expression on macrophages under different treatment. The data were normalized to 0H-Si (set as 1). *N*=4, ***p < 0.001; ****p < 0.0001.


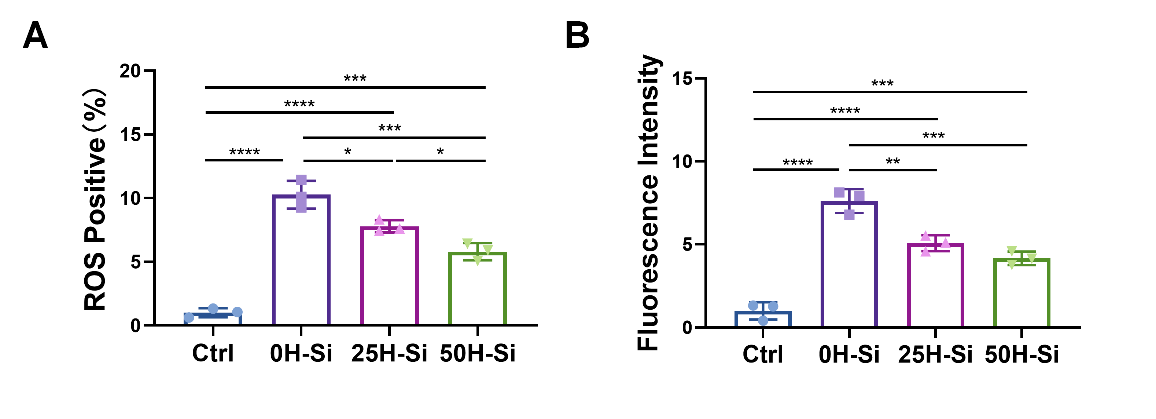


**Figure S5.** DCFH-DA fluorescent staining quantification of Raw264.7macrophages under different treatment. (**A**) Ratio of ROS positive Raw264.7 macrophages under different treatment. (**B**) Fluorescence intensity of Raw264.7 macrophages under different treatment. The data were normalized to Ctrl (set as 1). *N*=3, *p<0.05; **p<0.01; ***p < 0.001; ****p < 0.0001.


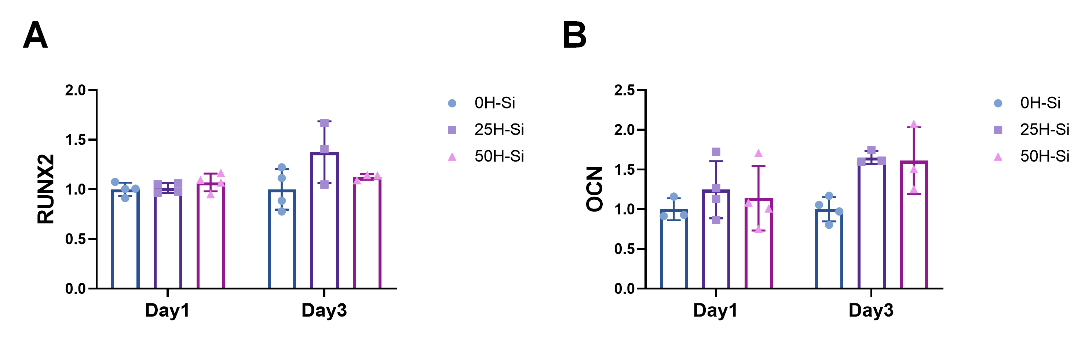


**Figure S6.** The expression level of osteogenic markers (**A**) *RUNX2* and (**B**) *OCN* in BMSCs at day 1 and 3. *N* = 3 or 4. Data were normalized to 0H-Si at day 1 (set as 1). No significant difference was seen between different groups.


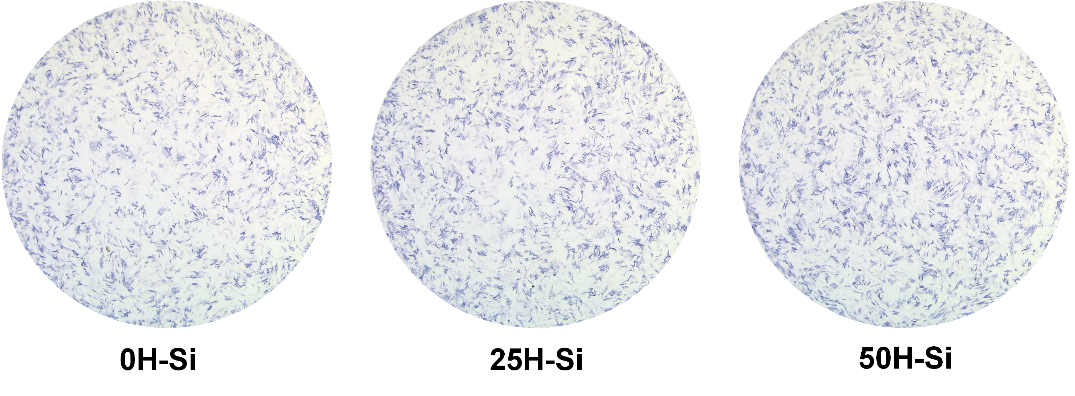


**Figure S7.** The alkaline phosphatase staining of BMSCs on Day 3. The BMSCs were incubated with TCP scaffold integrated with 0, 25 and 50 μg mL^-1^ H-Si in osteogenic medium for 3 d.


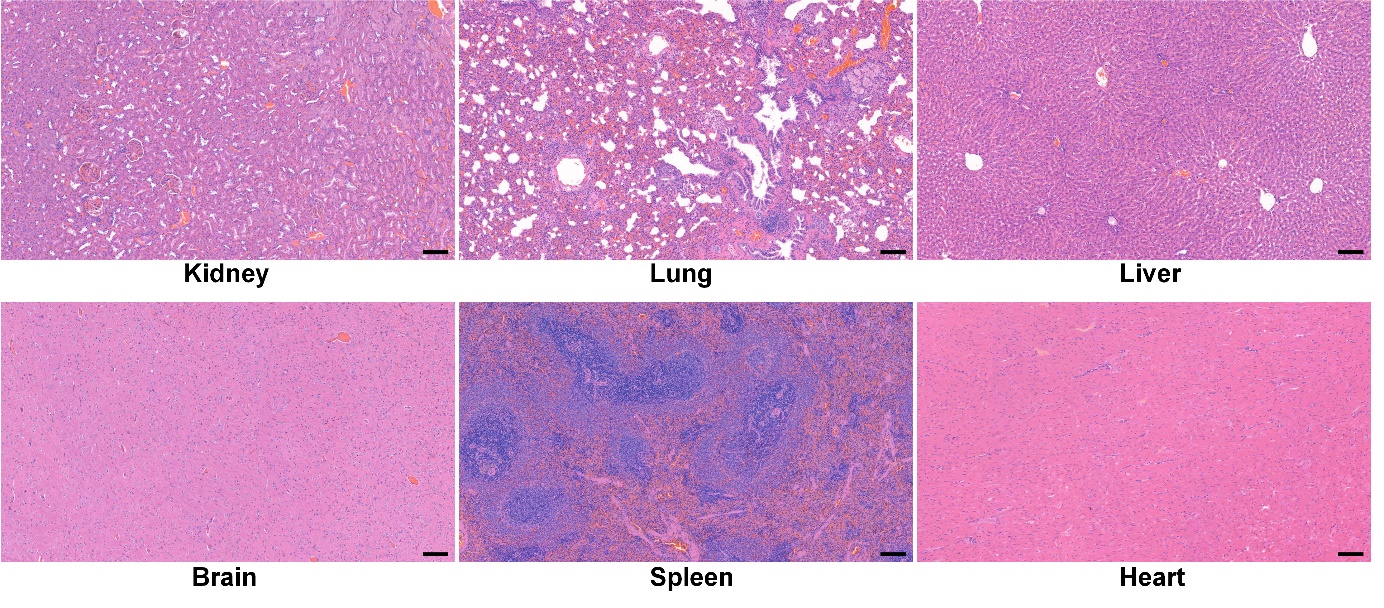
**Figure S8.** H&E staining of major organs of SD rats after 8 weeks of scaffolds *in vivo* implantation. Bar = 100 µm.


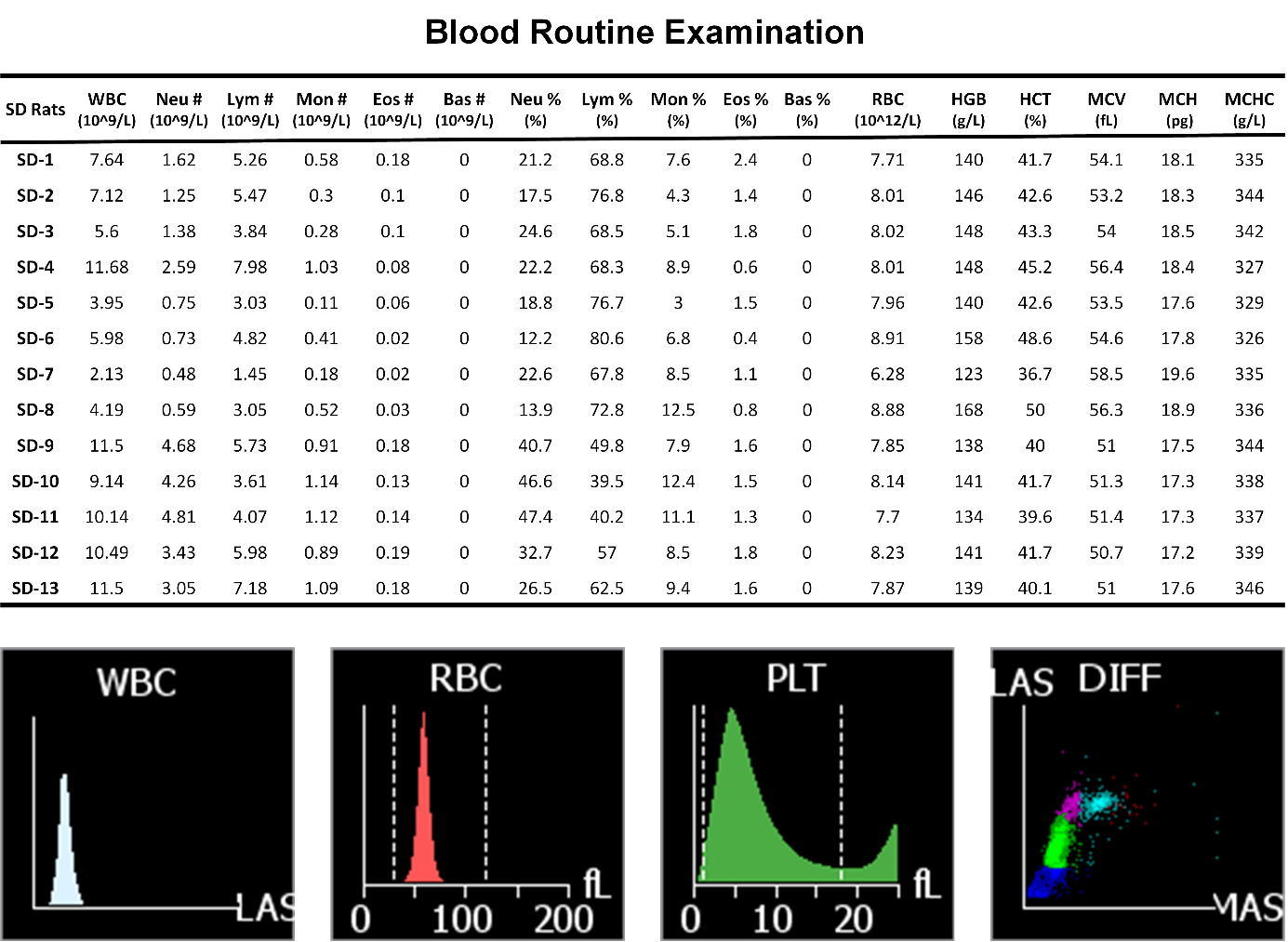


**Figure S9.** Blood routine examination of SD rats after 8 weeks of scaffolds *in vivo* implantation. Two blood samples of SD rats suffered severe hemolysis.


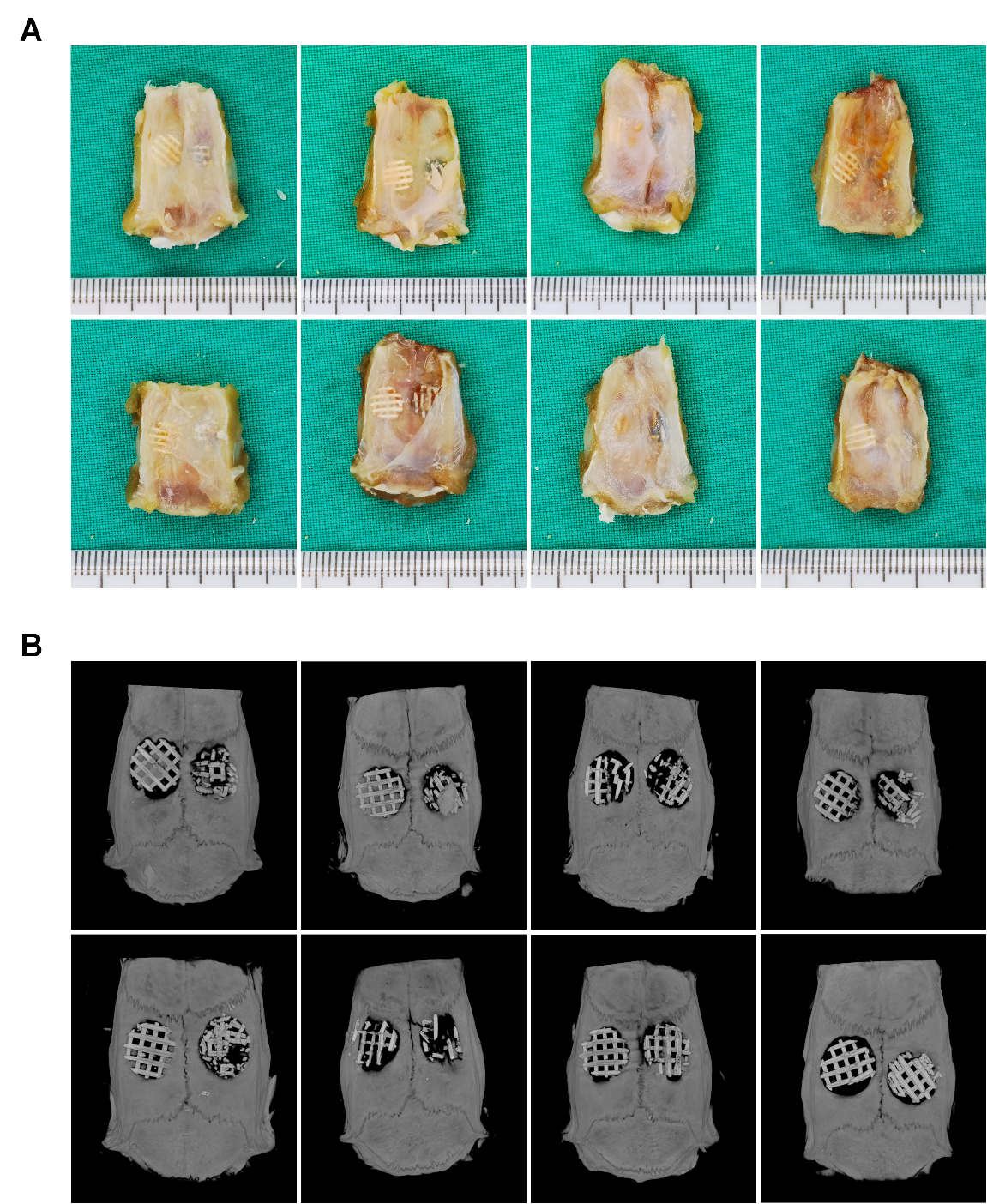


**Figure S10.** Scaffold residues in cranial defects. (**A**) Pictures and (**B**) 3D reconstruction of scaffold residues in cranial defects after 8 weeks of *in vivo* implantation. The TCP scaffolds were implanted into the left cranial defects, and the H-Si TCP scaffolds were implanted into the right cranial defects.


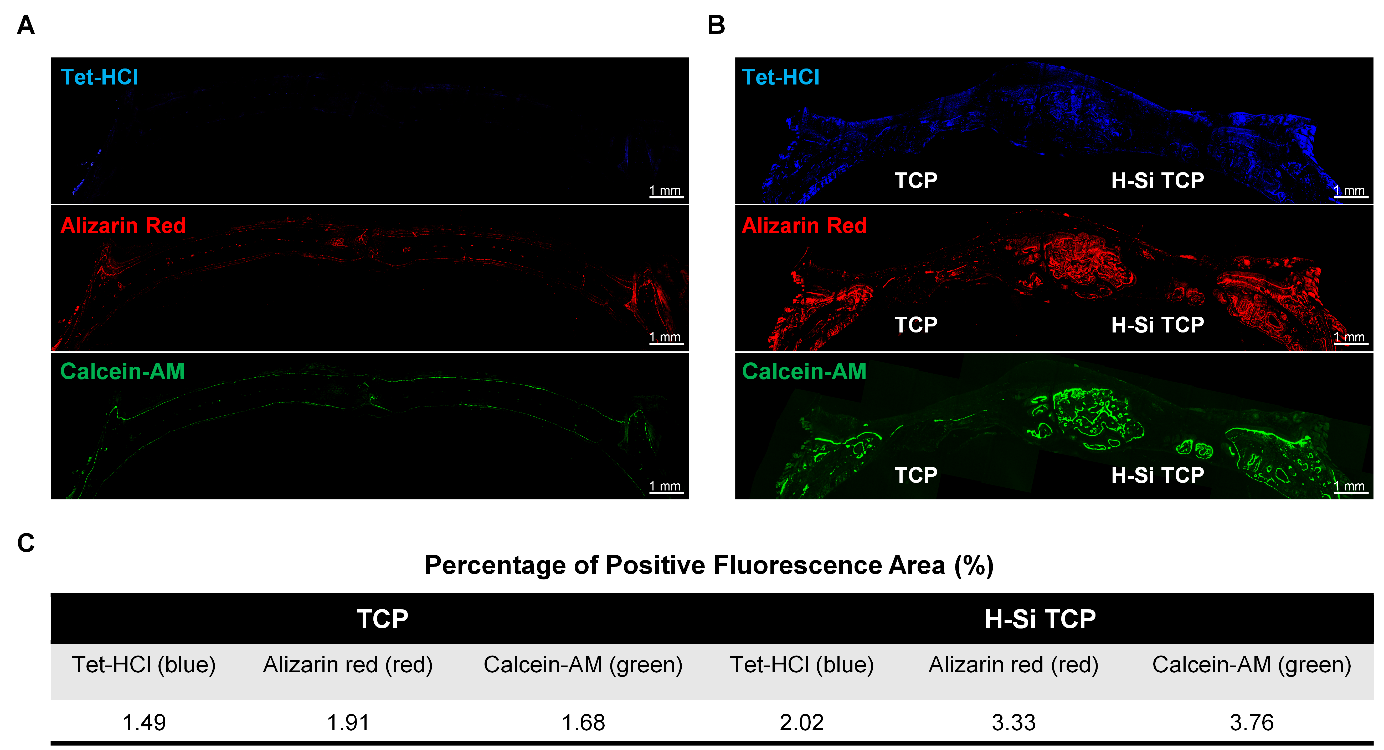
 **Figure S11.** Fluorescent imaging of intact and defective cranium in SD rats. (**A**) Fluorescent imaging of intact cranium. (**B**) Fluorescent imaging of defective cranium implanted with TCP and H-Si TCP scaffolds. Tet-HCl (blue fluorescence), alizarin red (red fluorescence), calcein (green fluorescence) were injected subcutaneously into SD rats at week 2, 4, and 6. (**C**) Quantification of each fluorescent area in cranial defect implanted with TCP or H-Si TCP scaffold.


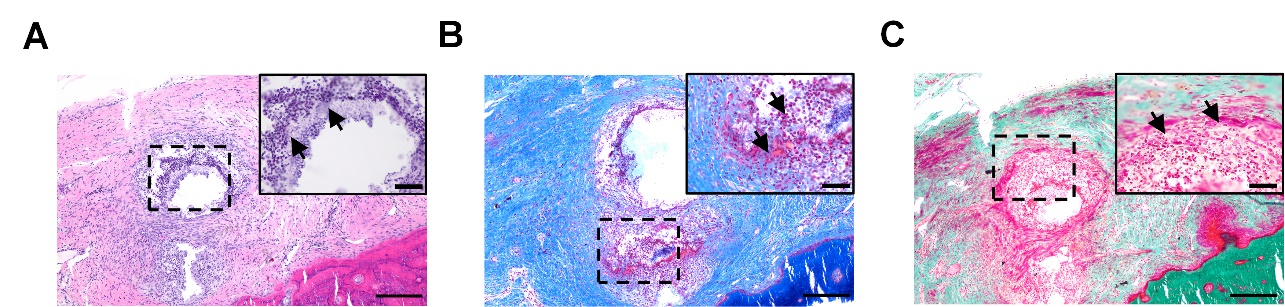


**Figure S12.** Histological staining of cranial bone defects implanted with TCP scaffolds. (**A**) H&E, (**B**) Masson trichrome and (**C**) Goldner trichrome staining of the cranial defects after 8 weeks of in vivo implantation. Residual TCP scaffold was surrounded by a large number of inflammatory cells, indicating the compromised conversion from inflammatory into regenerative status. The black arrows indicate surrounding the inflammatory cells. Bar=200μm and 50μm in lower and higher magnification.


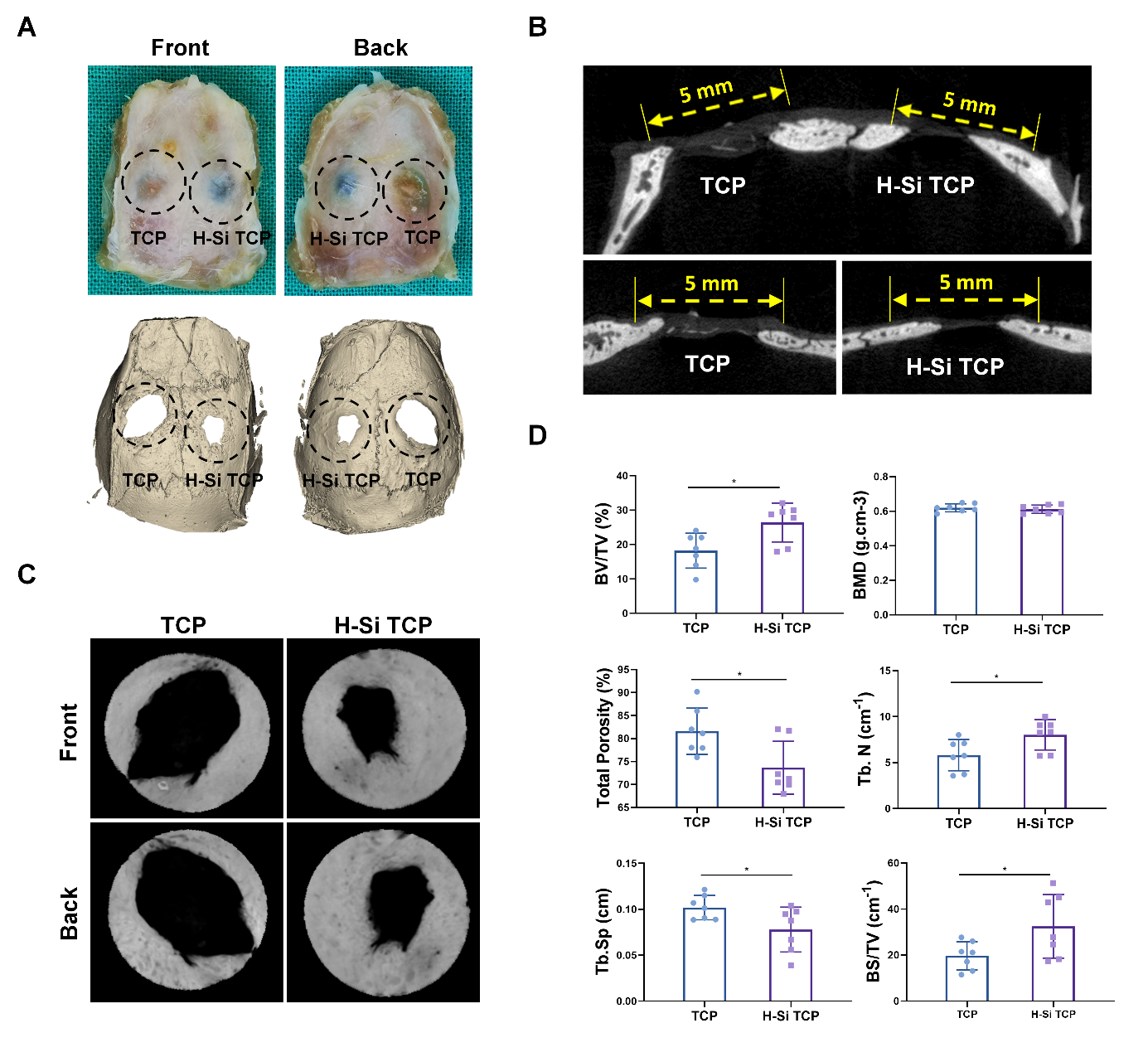


**Figure S13.** Micro-CT analysis of material-guided bone regeneration of TCP and H-Si TCP scaffolds in SD rats. (**A**) Typical pictures and 3D reconstruction of micro-CT scanning of cranial defects after 8 weeks of in vivo implantation. The scaffolds were completely degraded on both sides. (**B**) The transaxial (upper) and sagittal (lower) section of cranial defects implanted with TCP and H-Si TCP scaffolds. (**C**) 3D reconstruction of cranial defects implanted with TCP and H-Si TCP scaffolds. (**D**) Histomorphometric analysis of bone regeneration in cranial defects implanted with TCP and H-Si TCP scaffolds. *N*=7, *p < 0.05.


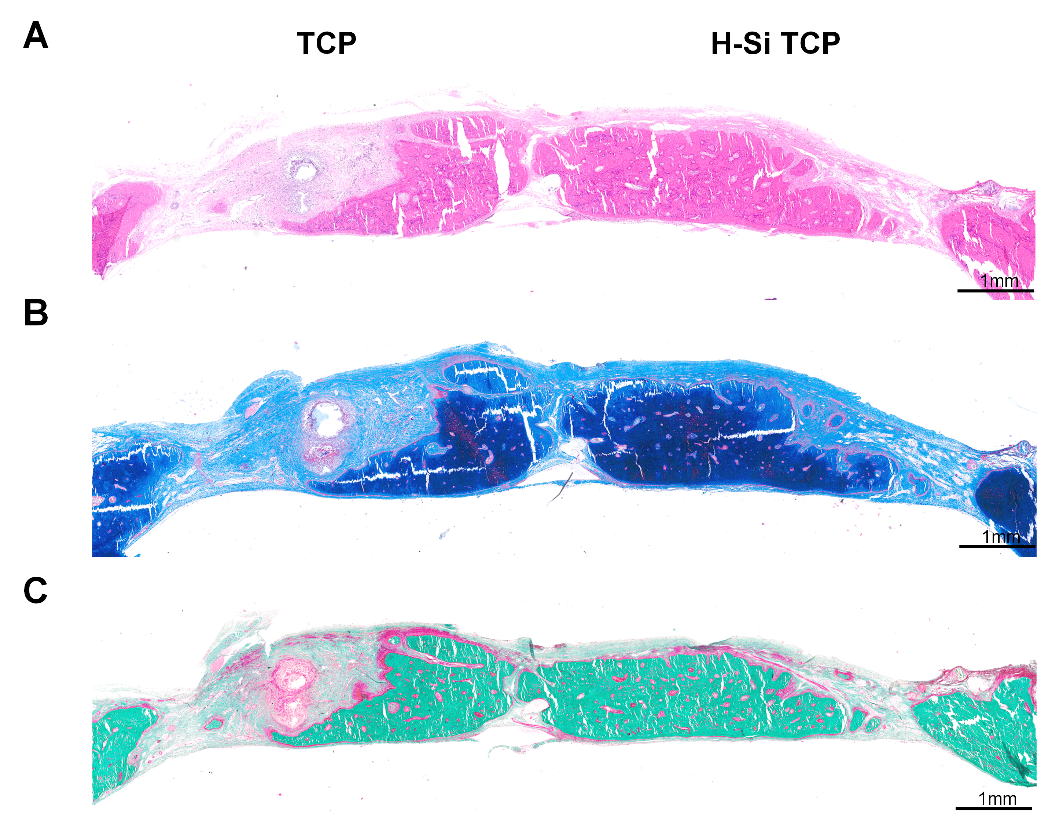


**Figure S14.** Histological staining of cranial bone defects implanted with TCP and H-Si TCP scaffolds. (**A**) H&E, (**B**) Masson trichrome and (**C**) Goldner trichrome staining of the cranial defects after 8 weeks of *in vivo* implantation.


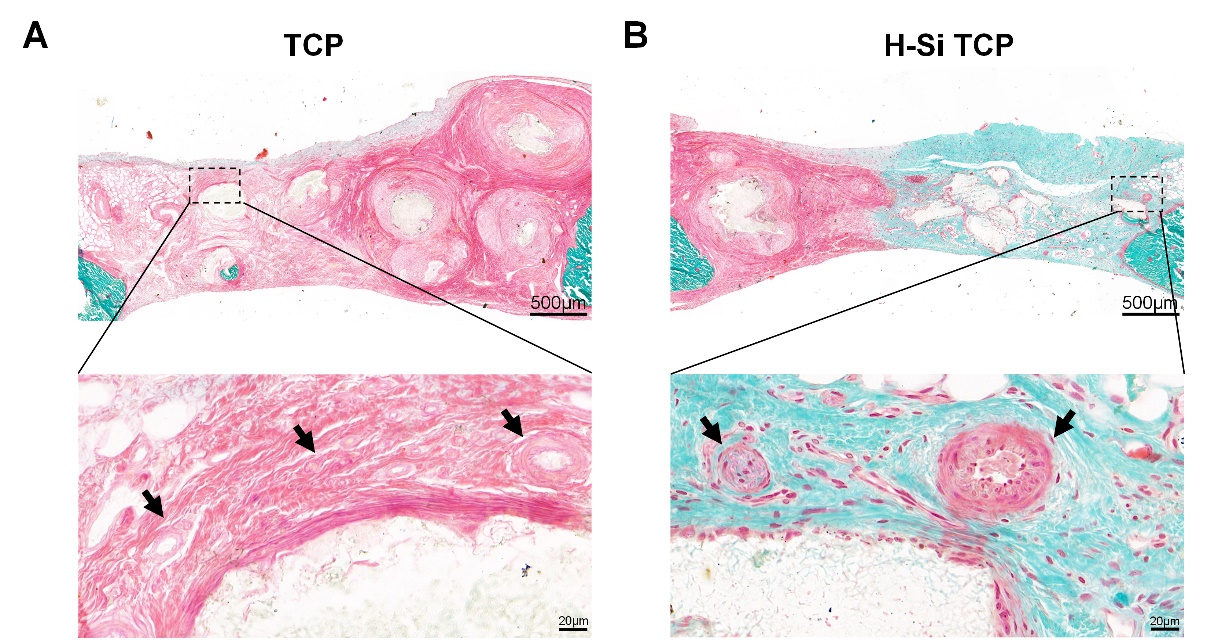


**Figure S15.** Neovascularization around the implanted scaffolds in cranial defects. (**A**) TCP scaffolds was surrounded by thick fibrous tissue in cranial defects. (**B**) Neovascularization was found around scaffold on H-Si TCP side.
